# Ultra‐Fast Label‐Free Serum Metabolic Diagnosis of Coronary Heart Disease via a Deep Stabilizer

**DOI:** 10.1002/advs.202101333

**Published:** 2021-07-29

**Authors:** Mengji Zhang, Lin Huang, Jing Yang, Wei Xu, Haiyang Su, Jing Cao, Qian Wang, Jun Pu, Kun Qian

**Affiliations:** ^1^ State Key Laboratory for Oncogenes and Related Genes School of Biomedical Engineering Institute of Medical Robotics and Med‐X Research Institute Shanghai Jiao Tong University Shanghai 200030 P. R. China; ^2^ State Key Laboratory for Oncogenes and Related Genes Division of Cardiology Renji Hospital School of Medicine Shanghai Jiao Tong University Shanghai Cancer Institute 160 Pujian Road Shanghai 200127 P. R. China

**Keywords:** deep learning, diagnostics, coronary heart diseases, mass spectrometry, metabolites

## Abstract

Although mass spectrometry (MS) of metabolites has the potential to provide real‐time monitoring of patient status for diagnostic purposes, the diagnostic application of MS is limited due to sample treatment and data quality/reproducibility. Here, the generation of a deep stabilizer for ultra‐fast, label‐free MS detection and the application of this method for serum metabolic diagnosis of coronary heart disease (CHD) are reported. Nanoparticle‐assisted laser desorption/ionization‐MS is used to achieve direct metabolic analysis of trace unprocessed serum in seconds. Furthermore, a deep stabilizer is constructed to map native MS results to high‐quality results obtained by established methods. Finally, using the newly developed protocol and diagnosis variation characteristic surface to characterize sensitivity/specificity and variation, CHD is diagnosed with advanced accuracy in a high‐throughput/speed manner. This work advances design of metabolic analysis tools for disease detection as it provides a direct label‐free, ultra‐fast, and stabilized platform for future protocol development in clinics.

## Graphical abstract

Mass spectrometry (MS) based metabolic analysis has the potential to provide real‐time monitoring of patient status for diagnostic purposes, whereas the diagnostic application is limited due to sample treatment and data quality/reproducibility. Here, the generation of a deep stabilizer is developed for ultra‐fast, label‐free MS detection in serum metabolic diagnosis of coronary heart disease.

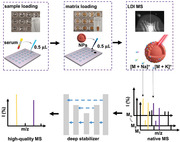

## 1 Introduction

Diagnostics is the key to precision medicine in the customization of healthcare for optimal treatment decisions,^[^ ^1^ ^]^ and two‐thirds of clinical diagnoses rely on in vitro diagnostics (IVD).^[^ ^2^ ^]^ Compared to the traditional analytical approaches (e.g., nuclear magnetic resonance (NMR) and biochemical methods), mass spectrometry (MS) demonstrates advantages of molecular identification capability and high analytical speed. Conventionally, NMR^[^ ^3^ ^]^ measures atomic species by their electromagnetic response from specific atoms in the interaction with magnetic fields and biochemical methods^[^ ^4^ ^]^ require enzymes or antibodies for targeted molecular recognition based selective reaction,^[^ ^4^, ^5^ ^]^ both of which usually need relatively long detection time due to the relaxation or recognition/reaction. For comparison, MS can directly record the mass‐to‐charge ratio (m/z) of molecules and their fragments, affording enhanced molecular identification capability and high analytical speed.

Notably, two major ionization mechanisms for MS are in clinical use: electrospray ionization (ESI) and laser desorption/ionization (LDI). These two mechanisms rely on ion or electron transfer in the primary ion formation during ionization,^[^ ^6^ ^],^ for example, absorption of photons with matrix for solid‐to‐gas transition in LDI.^[^ ^7^ ^]^ However, both of these types of MS require sample treatment that restricts the real‐world applications. Specifically, most clinical MS approaches require rigorous multi‐step chromatography and derivatization procedures to reduce sample complexity and enrich target molecules,^[^ ^8^ ^]^ but these procedures bring trade‐offs in terms of decreased speed/efficiency and increased sample consumption. Moreover, isotope‐labeling is commonly used as a sample pretreatment, inevitably increasing the expense (≈$2000/g) and expert labor/time (days per isotope synthesis and hours per isotope labeling) required for the diagnostic use of MS. The development of nanotechnology, enhancing charge transfer and decreasing heat dissipation for photon‐induced desorption/ionization of analytes in primary formation with nanomaterials as matrix,^[^ ^7^, ^9^ ^]^ has shed light on MS ionization mechanisms (especially for LDI‐MS), and may offer high performance in a label‐free manner without sample treatment. The rational design of a nanoparticle‐assisted MS method would serve as a potential ground‐breaking solution for IVD.^[^ ^10^ ^]^

The clinical use of MS depends on high data quality (referring to high‐reproducibility on diagnosis, indicated by diagnostic coefficient of variation (CV) < 10% (CV of predicted labels). Notably, a large number of technical replicates are needed to ensure high data quality and reproducibility.^[^ ^11^ ^]^ Whereas, to ensure high data quality and reproducibility for deep matrix‐assisted LDI‐MS, millions of laser shots are required.^[^ ^12^ ^]^ The acquisition of high‐quality results in this manner is expensive, time‐consuming, and labor‐intensive, which limits the large‐scale clinical applicability of this method. Data quality can be significantly enhanced by machine learning, particularly deep learning. Due to the task‐oriented learning strategy to encode features and intrinsic data representations via nonlinear modules,^[^ ^13^ ^]^ deep learning has been successfully applied to complex signal reconstruction tasks,^[^ ^14^ ^]^ such as low‐dose to normal‐dose computed tomography (CT) images transfer and magnetic resonance images to CT images transfer.^[^ ^15^ ^]^ Yet, despite recent successes in the high‐quality prediction of tandem MS (MS/MS) data,^[^ ^16^ ^]^ the applications of deep learning in MS is very limited in terms of both the acquisition of high‐quality MS data and subsequent diagnostic application.

We developed and applied our deep learning method in the context of disease diagnosis using serum metabolic profiles (SMPs), serum blueprints extracted from LDI‐MS results for distinguishing patients from controls. Metabolic disorder is associated with a majority of diseases, including coronary heart disease (CHD), which accounts for half of cardiovascular‐related deaths.^[^ ^17^ ^]^ CHD includes myocardial infarction (MI), which exhibits the highest mortality among CHD cases (47.8%), resulting in millions of deaths worldwide per year.^[^ ^17^ ^]^ Notably, speed is critical in the early detection of MI for emergency use to save patient lives and improve quality of life. The high‐sensitivity cardiac troponin I/T (cTnI/T) test is applied to almost every CHD patient with suspected MI. Despite its near ubiquitous use, the troponin assay requires the serial cTnI measurements (up to 9 h) and one measurement demands at least 15–30 min for the antibody–antigen recognition on which the method depends to occur.^[^ ^4^, ^18^ ^]^ Furthermore, for non‐MI CHD, troponin does not provide any valuable diagnostic information, and angiography plus electrocardiogram (the gold standard diagnostic method) is used instead, which can be invasive and is thus not practical for general screening.^[^ ^19^ ^]^ Therefore, improved methods to detect CHD, particularly both MI and non‐MI, are needed.

To address the major challenges described above, we applied generated ferrous nanoparticles (NPs) and a deep stabilizer and used them to develop an ultra‐fast, label‐ and antibody‐free MS‐based method for a stabilized metabolic diagnosis with trace serum. This method was verified by assessing its performance in diagnosing CHD (both MI and non‐MI CHD) and found to exhibit advantages over existing methods. Nanoparticle‐assisted LDI‐MS may represent a revolution in the detection of metabolic disorders and contribute to improving health care.

## 2 Results and Discussion

### 2.1 Metabolic Analysis of Trace Serum by Nanoparticle‐Assisted LDI‐MS

We performed ultra‐fast serum metabolic analysis by NP‐assisted LDI‐MS, with high‐throughput/speed solid phase ionization (solid‐to‐gas transition) enhanced by orders of magnitude over liquid phase ionization (liquid‐to‐gas transition) by ESI. LDI‐MS incorporated label‐free sample preparation and MS detection in 30 s, for an overall experimental time < 1 min per sample. Ferrous NPs were selected as the matrix for LDI‐MS, due to its low‐cost and straightforward synthesis procedure for large‐scale use.^[^ ^10d^ ^]^ We prepared ferrous NPs by wet chemistry, which yielded ≈0.83 g of product per batch (from three independent batches, Figure S1a, Supporting Information), a yield sufficient for 1,660,000 tests (0.5 µg/test). The ferrous NPs, where its ferrous oxide composition was indicated by energy‐dispersive X‐ray spectrum (Figure S1b, Supporting Information), had a rough surface (Figure S1c, Supporting Information) and crystalline structure (Figure S1d–f, Supporting Information), according to scanning electron microscopy, transmission electron microscopy, and selected area diffraction images. In addition, the ferrous NPs strongly absorbed light for transferring laser energy (Figure S1g, h, Supporting Information), and had good water dispersity adequate for their use as a matrix (Figure S1i, Supporting Information) and a negatively charged surface for cation adduction (Figure S1j, Supporting Information), as summarized in Figure S1k, Supporting Information. All the above characteristics, including low costs, rough surface, and related parameters, would be beneficial for LDI‐MS analysis.

In the absence of sample treatment, the successful metabolic analysis of a small volume of serum (0.5 µL) requires good salt and protein tolerance. Therefore, to assess the salt tolerance of ferrous NP‐assisted LDI‐MS detection, we first analyzed a mixture of three metabolites (valine, lysine, and glucose, see Figure S2a–c, Supporting Information, for standards) after cation adduction in 0.5 µL of highly concentrated solutions of NaCl (0.5 m, Figure S2d, Supporting Information) and KCl (0.5 m, Figure S2e, Supporting Information). In each case, we identified molecular peaks characteristic of the metabolites examined. Then, to assess the protein tolerance of ferrous NP‐assisted LDI‐MS detection, we analyzed the same three metabolites combined with bovine serum albumin at a high concentration (5 mg mL^−1^) and again obtained molecular peaks characteristic of the three metabolites (Figure S2f, Supporting Information). In addition, we performed the LDI MS detection results using the conventional organic matrices (*α*‐cyano‐4‐hydroxycinnamic acid (Figure S3a, Supporting Information) and 2,5‐dihydroxybenzoic (Figure S3b, Supporting Information)), showing both strong interference in low mass range^[^ ^10^, ^20^ ^]^ and limited sensitivity/selectivity^[^ ^10^, ^21^ ^]^ in the analysis of glucose (1 ng nL^−1^), compared to ferrous NPs (Figure S3c, Supporting Information). The above results highlighted the advantages of NP‐assisted LDI MS. Notably, our method allows a large number of tests (1000–2000) to be carried out in a very low analyte volume (≈µL), unlike conventional methods, which require a large volume (≈mL) and yield only a small number of tests (< 10).^[^ ^3^, ^8^, ^22^ ^]^ Moreover, the detection limits (≈µm, the lowest concentration can be detected) toward standard metabolites were of high sensitivity (Table S1, Supporting Information) considering the concentrations of metabolites in human serum (≈mm–µm).^[^ ^23^ ^]^ We therefore conclude that our ferrous NP‐assisted LDI‐MS achieved selective and sensitive LDI for diagnostic use.

Notably, clinical MS relies on rigorous multi‐step chromatography and derivatization procedures with slow analytical speed (up to hours per sample) and large sample volumes (on the order of milliliters) in order to reduce sample complexity by desalting and the removal of highly abundant proteins,^[^ ^8^, ^22^ ^]^ and thus enrich target molecules (see Table S2, Supporting Information, for a comparison among techniques). In contrast, NP‐assisted LDI‐MS requires no sample pretreatment, is fast (taking 30 s for analysis), and requires only a small sample volume (0.5 µL of serum as optimized, Figure S4, Supporting Information); both its analytical speed and required sample volume represent improvements of over one order of magnitude compared to clinical MS. Also, for sample storage, serum samples were stored at −80 degree in biobank for repeating use in diagnostics. Our methods may improve the reusability (0.5 µL per test) by 1–2 orders of magnitude affording minimum storage volume (≈µL), compared to prevailing diagnostic methods which requires large storage volume (≈mL).^[^ ^3^, ^8^, ^22^ ^]^ Therefore, our method addresses sample treatment, the key challenge to the universal use of clinical MS, as it requires no pretreatment procedures and only a very small sample volume.

### 2.2 Validation of Nanoparticle‐Assisted LDI‐MS via Diagnosis of MI and Non‐MI Patients Using SMPs

**Figure 1:**
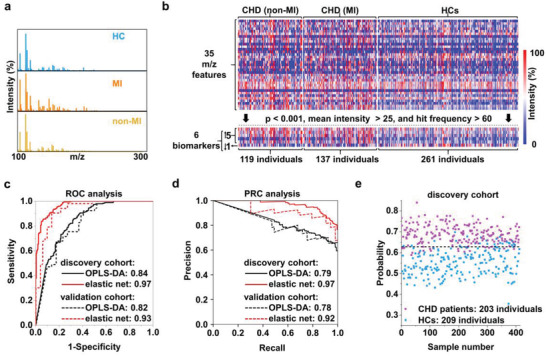
SMPs for machine learning. a) Typical MS spectra within a m/z range from 100 to 300 obtained by NP‐assisted LDI of serum samples from a HC, a CHD patient with MI and a CHD patient without MI. b) SMPs for HCs and CHD (MI/non‐MI) patients. Each SMP contained 35 m/z features from which six biomarkers (*p* < 0.001, mean intensity > 25, and hit frequency > 60) were screened. Specifically compared to HCs, five biomarkers were up‐regulated and one biomarker was down‐regulated in CHD patients. c–e) Diagnostic performance of machine learning for stratification and prediction. c) Receiver operating characteristic curves and d) PRC analysis using OPLS‐DA (black) and sparse learning (elastic net analysis, red) to distinguish HCs from CHD patients. The solid and dashed lines showed the results from the discovery and validation cohorts, respectively. e) Stratification based on the predicted probability for CHD patients and HCs obtained by sparse learning (elastic net analysis) of SMPs in the discovery cohort. Blue and purple points represented HCs and CHD patients, respectively. The dashed lines in (e) indicated the machine learning‐derived thresholds with an optimized AUC to distinguish CHD patients from HCs.

Considering the global demand of a speedy and non‐invasive tool for precision diagnosis of CHD toward efficient treatment, we conducted direct label‐free metabolic analysis of serum for CHD patients (CHDs, including MI and non‐MI) and healthy controls (HCs) in **Figure** **1**. All CHD patients, including MI and non‐MI patients, had a specific diagnosis by angiography and electrocardiography, and all clinical information for each case was reviewed by two pathologists without knowledge of the clinical course of the patient. For MI patients, 99th percentile cTnI levels were recorded by high‐sensitivity chemiluminescence immunoassay. Serum samples were also collected from the HCs, who had no clinical evidence of cardiovascular disease or other major diseases. Power analysis (a universal method to obtain the optimal sample size within a significant level of confidence) was performed on a pilot dataset of 16 samples (8/8, CHD/control) to compute the minimum sample number required for the meaningful machine learning (Figure S5, Supporting Information).^[^ ^24^ ^]^ Based on the result of power analysis, the minimum number of samples was 160 (80/80, CHD/control) for achieving the predicted power of > 0.8, which is significant to conclude the statistical meaningful results according to previous references.^[^ ^10^, ^25^ ^]^ Finally, 517 individuals were enrolled (414 individuals as the discovery cohort and 103 individuals as the validation cohort), of which 261 were HCs and 256 were CHD patients (137 MI patients and 119 non‐MI patients, **Table** **1** and Table S3, Supporting Information). There was no significant difference in age or sex between the HCs and CHD patients (Table S3, Supporting Information). Typical MS spectra obtained from a MI patient and non‐MI CHD patient and a HC are shown in Figure 1a, with differences hardly recognized by naked eyes.

**Table 1:** Comparison of diagnostic performances by no stabilization and deep‐stabilized experiments in the validation cohort

Patient category^a)^	Deep stabilizer^b)^	CV/AUC^c)^	CV/sensitivity^c)^	CV/specificity^c)^	Threshold^d)^	Sensitivity^d)^
CHD	N	12%/0.86	13%/0.81	14%/0.80	23rd	0.98
	D	3%/0.95	5%/0.91	5%/0.89	70th	0.98
MI	N	12%/0.85	13%/0.82	13%/0.79	22nd	0.98
	D	4%/0.95	5%/0.93	7%/0.88	69th	0.98
non‐MI	N	12%/0.86	14%/0.83	14%/0.81	24th	0.98
	D	3%/0.95	5%/0.93	6%/0.89	70th	0.98

^a)^ Patient category comprised coronary heart disease patients (denoted CHD), CHD patients with myocardial infarction (denoted MI), and CHD patients without myocardial infarction (denoted non‐MI).

^b)^ Deep stabilizer column indicated whether native MS results were deep‐stabilized (denoted D) or no stabilization (denoted N).

^c)^ CV referred to the coefficient of variation, which was calculated as the ratio of the standard deviation to the mean. AUC referred to the area under the receiver operating characteristic curve. Sensitivity referred to the ratio of the number of true positives to the total number of patients. Specificity referred to the ratio of the number of true negatives to the total number of controls. Sensitivity and specificity were determined by the upper‐left point in ROC plot based on machine learning.

^d)^ Threshold referred to the cut‐off for diagnosis, represented by the nth percentile upper reference probability of HCs, equivalent to specificity. Sensitivity was determined at CV of 10% according to the DVC‐derived thresholds.

We constructed label‐free SMPs (Figure 1b) for machine learning in a step‐wise manner (Figure S6, Supporting Information). Data processing consisted of four main steps: signal extraction by preprocessing, feature selection by signal‐to‐noise ratio (S/N), profile recognition by machine learning, and biomarker screening by multiple restrictions. In the initial signal extraction step, we performed preprocessing using the local maximum method to decrease the complexity of native MS results from ≈80,000 data points per spectrum before extraction) to 273 metabolite peaks after extraction. In the second step—feature selection—we excluded features with background S/N ≤ 3, and found 35 m/z features comprising the SMPs. In the third data processing step—profile recognition—we used machine learning to differentiate the SMPs from different groups, including CHD/MI/non‐MI. Finally, for biomarker screening, we used multiple restrictions (with a hit frequency > 60, mean intensity > 25, and *p* < 0.001) to choose the features in the SMP and identify m/z features that might represent metabolic biomarkers (Table S4, Supporting Information).

In the profile recognition step, we studied the diagnostic performance of two major machine learning algorithms: orthogonal projections to latent structures discriminant analysis (OPLS‐DA, a combined multivariate analysis as universally used in metabolic analysis) and elastic net analysis (a form of sparse learning) in Figure 1c and Table S5, Supporting Information.^[^ ^10^, ^26^ ^]^ For conventional OPLS‐DA, the diagnostic performance in distinguishing CHD patients from HCs was unsatisfactory (area under the curve, AUC < 0.9),^[^ ^27^ ^]^ with AUC/sensitivity/specificity values of 0.84 (0.80–0.88 at a 95% confidential interval (CI))/0.73/0.78 in the discovery cohort and 0.82 (0.74–0.90 at a 95% CI)/0.77/0.68 in the validation cohort (Figure 1c and Table S5, Supporting Information). In contrast, the sparse learning performed much better, with AUC/sensitivity/specificity values of 0.97 (0.95–0.98 at a 95% CI)/0.91/0.88 in the discovery cohort and 0.93 (0.88–0.98 at a 95% CI)/0.85/0.87 in the validation cohort (Figure 1c, Table S5, and Figure S8g, j, Supporting Information). Notably, we demonstrated similarly high diagnostic performance for distinguishing both MI (AUC of 0.97/0.92 for discovery/validation cohort) and non‐MI (AUC of 0.96/0.94 for discovery/validation cohort) patients from HCs (Figure S8, Supporting Information). We further performed precision‐recall curve (PRC) analysis^[^ ^28^ ^]^ on the diagnostic performances of sparse learning and OPLS‐DA. Based on the result of PRC, elastic net achieved higher area under PRC (AUPRC), with AUPRC increasing from 0.79/0.78 to 0.97/0.91 in the discovery/validation cohort for CHD diagnosis, respectively (Figure 1d and Figure S8d–f, Supporting Information).

To exclude the overfitting effects, we performed five‐fold cross‐validation (Figure S7, Supporting Information) and permutation test (Figure S9, Supporting Information). We performed the cross‐validation process in the discovery cohort and the independent validation cohort was used for blind test (Figure S7a, Supporting Information). Based on the diagnostic model with cross‐validation, no significant difference of AUC was found between cross‐validation and testing in every round according to the independent‐samples *t*‐test (*p* of 0.07–0.89, Table S6, Supporting Information). For permutation test, the classifier showed no overfitting effect with *p* < 0.05 from the distribution of the AUC calculated using the uninformative data obtained by random permutation (Figure S9, Supporting Information), which is universally employed to estimate overfitting.^[^ ^29^ ^]^ In addition, the consistency of diagnostic performance in both validation cohort (AUC of 0.91, blind test dataset in classifier testing) and discovery cohort (AUC of 0.93, cross‐validation in classifier training) further guaranteed a robust model without overfitting, according to previous reports.^[^ ^30^ ^]^

The superior performance of sparse learning over that of conventional OPLS‐DA may be due to two major factors. First, the MS data exhibits intrinsic sparsity; only a few biomarkers are potentially useful for diagnosis,^[^ ^10c–f^ ^]^ reflected in the finding that only six metabolite peaks were hit stably (hit frequency > 60) and were of significant intensity (*p* value less than 0.001, Table S4 and Figure S6b, Supporting Information). The second factor is that sparse regularization allows the classification contributions of these metabolite peaks to be gauged and high weights to be assigned to a limited number of biomarkers with relatively high importance.

### 2.3 Generation of a Deep Stabilizer for Enhanced MS Data and Diagnosis

**Figure 2:**
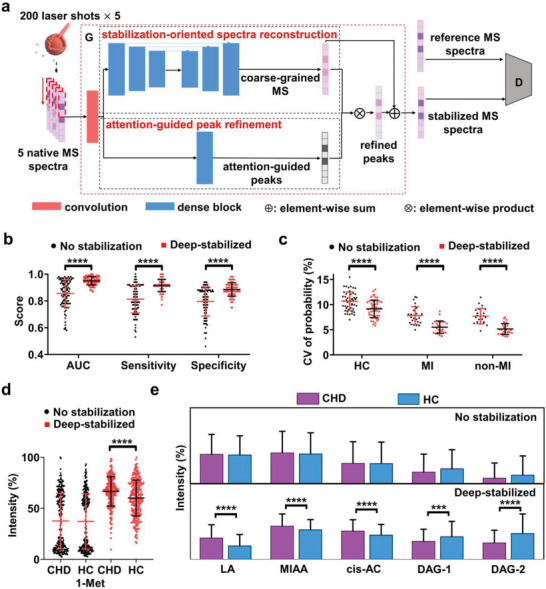
Construction and performance of the deep stabilizer. a) The generative adversarial network structure of the deep stabilizer, which included a generator (G) and discriminator (D). The generator used native MS results as input to produce stabilized MS results through two branches: stabilization‐oriented spectrum reconstruction and attention‐guided peak refinement. A convolutional layer was applied to the two branches (see Experimental Section). The final reconstruction used the attention‐guided peaks and coarse‐grained MS spectra to efficiently reconstruct refined peaks and stabilize the MS results through element‐wise production and addition. The discriminator calculated the probability of the stabilized MS results as the reference MS results. Blue rectangle represented the dense block as the basic feature extraction unit for both branches and the solid arrow referred to the forward feed direction. b) Diagnostic performance (AUC, sensitivity, and specificity) of no stabilization and deep‐stabilized data for the prediction of CHD in the validation cohort. c) CVs of predicted probabilities for HCs and CHD (MI and non‐MI) patients in the validation cohort through no stabilization and deep‐stabilized experiments. d) Data on levels of 1‐methylpyrrole (1‐met) in HCs and CHD patients obtained through no stabilization and deep‐stabilized experiments. e) The newly screened five biomarkers including LA, MIAA, cis‐AC, diacylglycerol (14:1/24:1) (DAG‐1), and diacylglycerol (24:1/20:4) (DAG‐2) through no stabilization and deep‐stabilized experiments. *** indicated *p* < 0.001 and **** indicated *p* < 0.0001 in paired‐samples *t*‐test (b,c) and independent‐samples *t*‐test (d,e).

We observed performance variation that affected the diagnostic sensitivity and specificity, thus sought to develop a method to improve accuracy and stabilize performance (**Figure** **2**a). To establish how this variation might affect the method's diagnostic use, we conducted 10 independent technical replicates on one sample from each of the 517 individuals in the two cohorts, with 1000 laser shots per replicate (Table 1). We observed a high level of variation in the data, with a CV of 12–14% for AUC, sensitivity, and specificity among the 10 replicates (Table 1). For example, in 100 blinded inter‐replication combinations of groups for the discovery and validation cohorts, the AUC ranged from 0.58 to 0.99—this unsatisfactory reproducibility (CV > 10%) would limit its practical use (Table 1 and Tables S7 and S8, Supporting Information). Notably, the overall AUC reached 0.98 across 10 replicates (corresponding to 10,000 laser shots per individual, Figure S6a, Supporting Information), which could enhance data quality and reduce variation.^[^ ^12^ ^]^ These results revealed the existence of variation in native MS data and demonstrated that high‐quality MS data were obtained through replication.

Due to the infeasible time and expense of replication in real‐case application, deep learning can produce high‐quality data with the task‐oriented learning strategy, to encode features and intrinsic data representations via nonlinear modules in a practical manner. Accordingly, we constructed a deep stabilizer to reduce variation in the MS data and thereby increase the accuracy of CHD diagnosis. Using the deep stabilizer, we mapped native MS data obtained from a small number of tests (200–1000 shots) to high‐quality data corresponding to a large number of tests (up to 10,000 shots; Figure 2a and Figure S10, Supporting Information). The deep stabilizer network consisted of two branches: one reconstructed high‐quality MS output from the low‐quality MS input, and the other acted as an attention mechanism that refined the signal peaks (Figure S11, Supporting Information). In order to control the diagnostic variation caused by the non‐predictable LDI process of complicated ionization models as involved, for example, lucky survivor model,^[^ ^31^ ^]^ and because a large number of tests can deliver high‐quality results for diagnosis, the stabilizer is expected to transform native MS data obtained from a low number of laser shots to enhanced MS data equivalent to that obtained from a large number of shots and thus stabilize the diagnostic performance.

Next, we tested the performance of the deep stabilizer by comparison of diagnostic variations. After processing with the deep stabilizer, the CVs for AUC, sensitivity, and specificity in the diagnosis of CHD were significantly (*p* < 0.0001) decreased to 3–5% (Figure 2b, Table 1, and Table S11, Supporting Information), while the mean AUC and sensitivity/specificity increased to 0.95 and 0.91/0.89 in the validation cohort, respectively. For comparison, in the same data before processing with the deep stabilizer, we observed CVs of 12–14% in the AUC and sensitivity/specificity, which reached only 0.86 and 0.81/0.80, respectively. Accordingly, we observed that the CV of predicted probabilities for each individual was significantly decreased for both MI and non‐MI patients in the validation cohort (Figure 2c), from 8 to 5%, after stabilizing. We observed consistent results in the discovery cohort (Figure S12 and Table S12, Supporting Information). We also studied the diagnostic performance with different numbers of laser shots when data were obtained with the deep stabilizer (Figure S13, Supporting Information). These data demonstrated enhanced diagnostic performance that was increased with an increasing number of laser shots with the deep stabilizer due to increased accuracy. Consequently, the deep stabilizer delivered remarkable performance in reducing the CV for the deep‐stabilized data.

We also investigated in detail the effect of data stabilization on the detection of metabolic biomarkers from the SMPs. For instance, in data that had not been processed with the deep stabilizer, there was no significant difference between the HCs and CHD patients in 1‐methylpyrrole level, which correlated with CHD (*p* = 0.87, Figure 2d and Table S4, Supporting Information). In contrast, after deep stabilization, we observed a significant difference between these participant groups (*p* < 0.0001), reflecting the upregulation of 1‐methylpyrrole in CHD patients that were in accord with results in Table S4, Supporting Information. More importantly, we newly screened 5 m/z features including lactic acid (LA), methylimidazoleacetic acid (MIAA), cis‐aconitic acid (cis‐AC), diacylglycerol (14:1/24:1), and diacylglycerol (24:1/20:4) as potential metabolic biomarkers, which showed significant differences only after deep stabilization (Figure 2e and Table S13, Supporting Information). Therefore, based on these data, we conclude that reconstruction of the biomarkers in the SMPs enhanced diagnostic performance, validating the effect of the deep stabilizer in detail.

### 2.4 Construction of Diagnosis Variation Characteristic Surface (Mengji‐Kun Surface)

**Figure 3:**
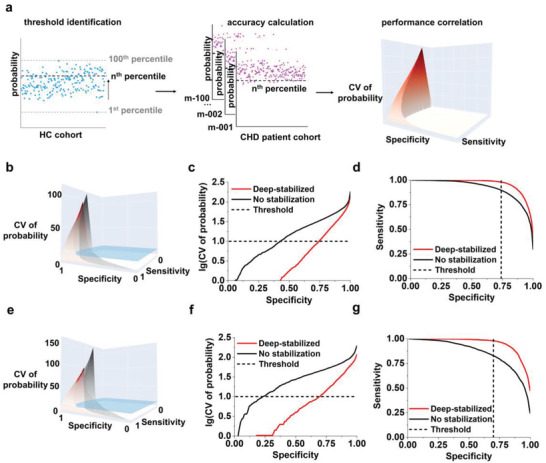
Establishment of a diagnostic protocol for CHD. a) Schematic of the diagnostic protocol based on the SMPs and machine learning. The protocol implemented three major procedures to obtain the DVC surface (here named the MK surface): threshold identification, accuracy calculation, and performance correlation. Application of the protocol in the b–d) discovery cohort and e–g) validation cohort. b,e) A 3D model showing MK surfaces for the indicated cohort. The blue plane represented the threshold for a CV of 10%. The grey and red MK surfaces referred to the no stabilization and deep‐stabilized experiments, respectively. Projections of MK surfaces showing c,f) CV/specificity and d,g) sensitivity/specificity were shown. The dashed line indicated the threshold for a CV of 10% according to the guidelines.

Considering the enhanced diagnostic performance (reduced CV and improved AUC) in CHD screening brought by the deep stabilizer, we sought to construct a novel tool to evaluate diagnostic sensitivity, specificity, and variation simultaneously. We established a diagnostic model for CHD by building a novel diagnosis variation characteristic (DVC) surface (also named as the Mengji‐Kun (MK) surface) using the SMPs and deep stabilizer (**Figure** **3**a and Table 1). The model building consisted of three major procedures: threshold identification with the threshold representing the percentile to distinguish CHDs from HCs, accuracy calculation with the accuracy referring to the probability variation among a series of technical replicates at the given threshold, and performance correlation with the performance including CV of probability as a function of sensitivity and specificity (Figure 3a).

Specifically, for threshold identification, we screened the nth percentile (from 1st to 100th percentile with a step of one percentile) upper reference predicted probability of the HC cohort as the threshold. The predicted probability of the HC cohort was obtained from the SMPs by sparse learning and the percentile was equivalent to specificity. For accuracy calculation, we measured the predicted probabilities of each CHD patient using 100 models, considering variation among inter‐replications. The nth percentile was applied in 100 models as the threshold to obtain 100 predicted labels for accuracy calculation. For performance correlation, we plotted the CV of probability as a function of diagnostic performance (sensitivity/specificity), generating a 3D model representing the MK surface. In this 3D model, the optimized diagnostic tool is expected to afford the minimum volume under the surface (VUS), indicating maximum accuracy and precision.

By applying this 3D model to the discovery cohort, we obtained a VUS of 1.14 using the deep stabilizer, but a less favorable VUS of 2.67 without the deep stabilizer (Figure 3b). Specifically, given a CV of < 10%, as recommended in the current guidelines for CHD diagnosis,^[^ ^11^ ^]^ the diagnostic performance with data stabilization reached the threshold for 74th percentile (Figure 3c and Figure S14a, Supporting Information) with a sensitivity of 0.98 (Figure 3d), a threshold that is superior to that of that obtained without data stabilization (43rd percentile with a sensitivity of 0.98, Figure S14b, Supporting Information). Similarly, in the validation cohort, we observed a VUS of 1.66 using the deep stabilizer and a VUS of 6.01 without the data stabilizer (Figure 3e), and the use of the data stabilizer enhanced the diagnostic performance threshold (from the 23rd to the 70th percentile) (Figure 3f and Figure S15, Supporting Information) with a sensitivity of 0.98 using deep‐stabilized data (Figure 3g) at a given CV of < 10%. Therefore, using the MK surface and VUS to evaluate diagnostic performance, we successfully validated our diagnostic protocol for CHD using the SMPs and deep stabilizer.

### 2.5 Establishment of a New Diagnostic Protocol for CHD

**Figure 4:**
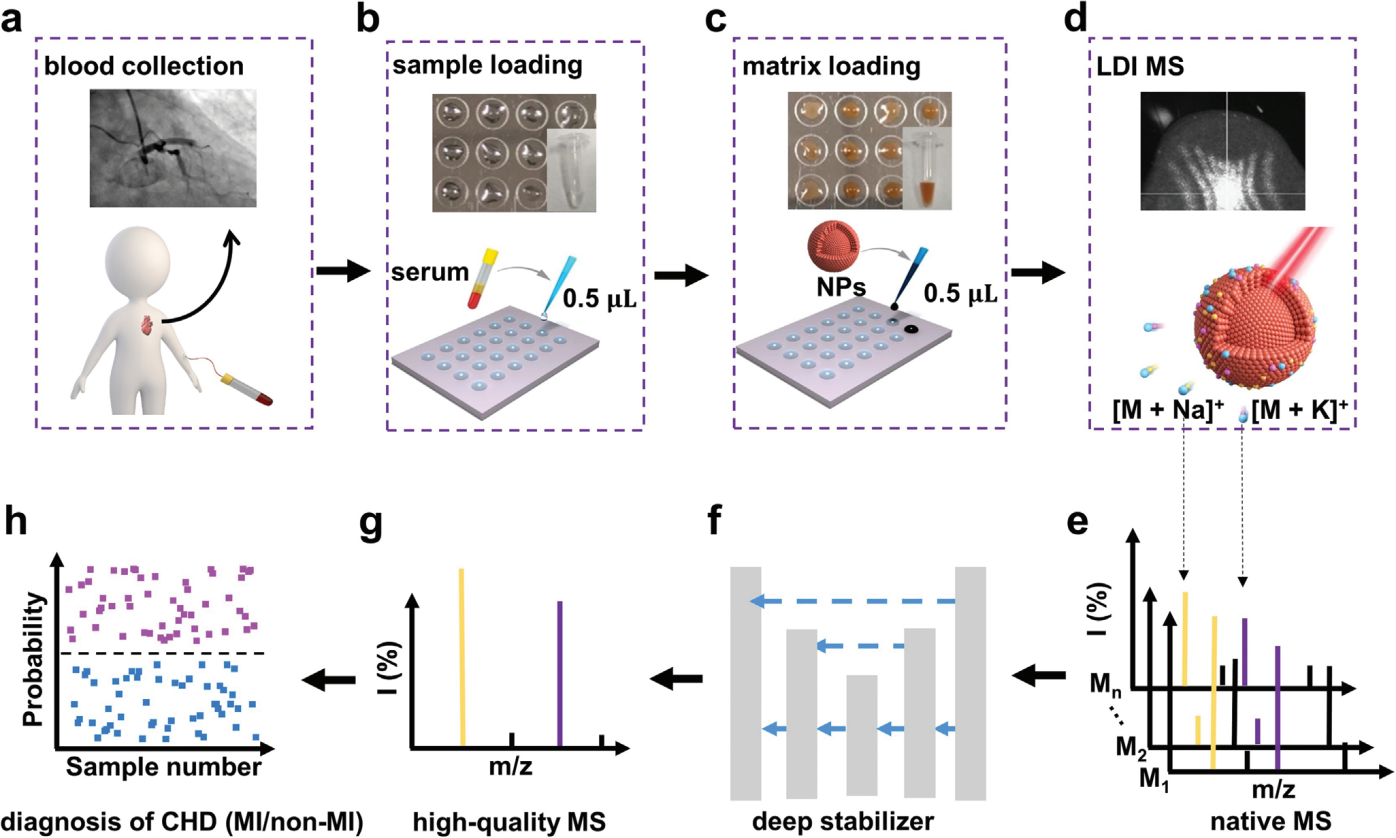
Schematic for an ultra‐fast, label‐ and antibody‐free serum metabolic diagnostic protocol. The protocol comprised two major phases: a–d) sample preparation (outlined in dashed lines) and e–h) data analysis. For the sample preparation phase, after a) sample collection from patients/controls, b) a volume of just 0.5 µL of native serum was directly loaded in a microarray, without any prior labeling, derivatization, or chromatography. Then, c) a suspension of ferrous NPs acting as a matrix is loaded on the microarray, followed by d) LDI to obtain cation adducts. For the data analysis phase, e) a given number of measurements (M1‐Mn) were obtained from native MS spectra for each individual and f) entered the deep stabilizer for signal reconstruction to predict high‐quality MS data in (g). Finally, h) these high‐quality MS data were further processed by sparse machine learning for the diagnosis of disease.

We have developed a novel protocol for the diagnosis of CHD using the SMP and novel data stabilizer (**Figure** **4**). Importantly, our serum metabolic approach has several advantages over the universal gold standard methods for the diagnosis of CHD (MI/non‐MI) patients (troponin assays, electrocardiography, and angiography).^[^ ^19^, ^32^ ^]^ The application of troponin assays is limited to CHD patients with MI, and fails to identify early‐stage CHD; in addition, each assay test requires at least 15–30 min for completion of the antibody reaction.^[^ ^4^, ^18^ ^]^ Electrocardiography requires slight visual changes in the electrocardiogram to be interpreted, and thus it can only be performed by experts with years of experience and it suffers from inter‐observer variability.^[^ ^32^, ^33^ ^]^ Finally, angiography is invasive and complicated to perform, prohibiting its use for general screening.^[^ ^32^ ^]^ In contrast to these procedures, our metabolic approach 1) achieved high diagnostic performance (including AUC, sensitivity, and specificity) in distinguishing CHD patients (both MI and non‐MI patients) from HCs; 2) facilitated ultra‐fast and label‐ and antibody‐free detection in seconds, with an experimental time less than 1 min toward on‐site and real‐time diagnosis; and 3) was minimally invasive with the use of blood samples. Therefore, NP‐assisted LDI‐MS could easily be applied on a large scale and make changes in cardiovascular clinics.

Typically, the establishment of a novel protocol is the key for a diagnostic assay that serves as a breakthrough and its universal clinical application. State‐of‐the‐art analytical tools for the diagnosis of CHD have evolved from blood glucose to cardiac troponin assays;^[^ ^11^, ^32^, ^33^, ^34^ ^]^ these assays were still in wide use and have had a large impact by efficiently reducing the morbidity and mortality of patients with CHD. For cardiac troponin assays, the diagnostic protocol affords the 99th percentile as the threshold for positivity with a sensitivity of 0.93–1.00 at the given CV of < 10%, but this assay cannot be applied to non‐MI CHD patients, as it detects elevated cTnI levels, which arise from impaired cardiac muscle due to MI.^[^ ^18^, ^32^ ^]^ Meanwhile, the blood glucose assay is applied to nearly every suspected CHD patient, but the diagnostic application of this assay is limited because glucose levels can also be elevated in many other circumstances, such as in response to diet in general and common diseases concurrent with CHD (e.g., diabetes).^[^ ^34^, ^35^ ^]^ Finally, blood lipoprotein levels, which form the basis of another typical CHD assay, are poorly specific and heritable, and thus vary widely among individuals.^[^ ^34^, ^36^ ^]^ Importantly, all the above assays require a certain number (≈4–8) of serial measurements (up to 9 h) to improve the sensitivity and the threshold used to diagnose CHD, while biomarker concentrations are measured.^[^ ^18^, ^35^ ^]^

The information given by the detection of SMPs using our deep stabilization method is more systematic than that obtained by these standard assays, providing a more comprehensive indication of an individual's disease status. Indeed, the desirable sensitivity and specificity of SMP detection allow the diagnosis of CHD (both MI and non‐MI). Furthermore, our protocol for the diagnosis of CHD (both MI and non‐MI) affords the 74th percentile as the threshold for positivity with a sensitivity of 0.98 and CV < 10%, due to deep‐stabilization of data reducing the CV and improving the specificity. Accordingly, our newly defined MK surface and the VUS can be used with our protocol to study diagnostic performance toward other disease detection to evaluate its performance, including the sensitivity, specificity, and CV. Hence, our protocol based on deep stabilization to obtain SMPs may function as a next‐generation diagnostic tool for CHD, similar to the use of cardiac troponin assays for the diagnosis of MI.

As a work of artificial intelligence assisting the screening of CHD, we expected two major research directions to be explored following this work, toward both medical science and computer science. For medical science, more CHD subtypes and control diseases would be included to demonstrate the universal application of our approach. For computer science, database construction would be desirable to maximize the overall performance of selected algorithms.

## 3 Conclusion

In summary, we generated ferrous NPs and applied them in NP‐assisted LDI‐MS, and further constructed a deep stabilizer to generate high‐quality MS results relating to SMPs from which biomarkers can be identified, toward better understanding of pathological process for early prevention, in‐time detection, and efficient control; these SMPs and the deep stabilizer were used to construct a novel method for the enhanced diagnosis of CHD. Our work represents several advances over currently available diagnostic protocols, for example, high analytical speed, antibody‐ and label‐free process, and detection of non‐MI patients, which cannot be achieved by traditional assays like troponin tests.

Our work will not only facilitate precision medicine for the diagnosis of CHD, but also lead to the development of personalized diagnostic tools for other metabolic diseases in the near future. Compared to conventional metabolic analytical approaches,^[^ ^2^, ^3^, ^22^ ^]^ our method may achieve millions of tests in common laboratory conditions by single mass spectrometer per year at low cost and high throughput, with advanced performance to study huge cohorts in large‐scale translational medicine.

## Conflict of Interest

The authors declare competing financial interest. The authors have filed patents for both the technology and the use of the technology to detect bio‐samples.

## Supporting information

Supporting Information

## Data Availability

The data that support the findings of this study are available from the corresponding author upon reasonable request.
