# Supplementary material for: Ultra‐Fast Label‐Free Serum Metabolic Diagnosis of Coronary Heart Disease via a Deep Stabilizer
Source: Adv Sci (Weinh). 2021 Jul 29;8(18):2101333. doi: 10.1002/advs.202101333 (PMC8456274; doi:10.1002/advs.202101333)
Supplement: Supplementary file 1 — Supporting Information [file ADVS-8-2101333-s001.pdf]

© 2021 Wiley-VCH GmbH

ADVANCED SCIENCE
Open Access

Supporting Information

for *Adv. Sci.*, DOI: 10.1002/advs.202101333

Ultra-fast label-free serum metabolic diagnosis of coronary heart disease via a deep stabilizer

*Mengji Zhang, Lin Huang, Jing Yang, Wei Xu, Haiyang Su, Jing Cao, Qian Wang, Jun Pu, and Kun Qian**

Supporting Information

Ultra-fast label-free serum metabolic diagnosis of coronary heart disease via a deep stabilizer

*Mengji Zhang, Lin Huang, Jing Yang, Wei Xu, Haiyang Su, Jing Cao, Qian Wang, Jun Pu, and Kun Qian**

Mengji Zhang, Dr. Lin Huang, Jing Yang, Wei Xu, Dr. Haiyang Su, Jing Cao, Prof. Qian Wang, and Prof. Kun Qian
State Key Laboratory for Oncogenes and Related Genes, School of Biomedical Engineering, Shanghai Jiao Tong University, Shanghai, 200030, P. R. China
State Key Laboratory for Oncogenes and Related Genes, Division of Cardiology, Renji Hospital, School of Medicine, Shanghai Jiao Tong University, Shanghai Cancer Institute, 160 Pujian Road, Shanghai, 200127, P. R. China
E-mail: k.qian@sjtu.edu.cn

Prof. Jun Pu
State Key Laboratory for Oncogenes and Related Genes, Division of Cardiology, Renji Hospital, School of Medicine, Shanghai Jiao Tong University, Shanghai Cancer Institute, 160 Pujian Road, Shanghai, 200127, P. R. China

This section includes the following:

1. Experimental Section
2. FigureS1-15
3. Table S1-13

Experimental Section

Chemicals and reagents

For the synthesis of ferrous nanoparticles, trisodium citrate(99.5%), ethylene glycol (99%), iron chloride hexahydrate (99%), sodium acetate anhydrous (99%), and ethanol absolute (99.7%) were purchased from Sinopharm Chemical Reagent Beijing Co., Ltd. (Beijing, China).L-lysine (98%), L-valine (98%),D-glucose (99.5%), L-methionine (98%), L-leucine (98%), L-arginine (99.5%), L-serine (99%), L-tyrosine (98%), L-alanine (98.5%), L-proline (98.5%),bovine serum albumin (BSA, 98%), α-cyano-4-hydroxycinnamic acid (CHCA, 99%), and 2,5-dihydroxybenzoic acid (DHB, 99%) were purchased from Sigma-Aldrich (St. Louis, MO, USA). Sodium chloride (99.5%) and potassium chloride (99.5%) were purchased from Sinopharm Chemical Reagent Beijing Co., Ltd. All aqueous solutions and suspensions used in the experiments throughout this work were prepared using deionized water (18.2 MΩ·cm, Milli-Q, Millipore, Molsheim, France).

Preparation and characterization of ferrous nanoparticles

Ferrous nanoparticles were synthesized by the following modified co-precipitation method.[1] Briefly, sodium citrate, sodium acetate, and ferric chloride hexahydrate were dissolved in 100 mL of ethylene glycol under vigorous stirring for 30 min. The mixture was then poured into a Teflon-lined stainless-steel autoclave and heated at 200°C for 10 h. After the reaction, the samples were carefully removed from

the autoclave and cooled to room temperature. The as-prepared products were thoroughly and alternately washed with ethanol and water, and finally dried at 60°C before use.

To characterize the ferrous nanoparticles, scanning electron microscopy (SEM) images and energy-dispersive X-ray (EDX) spectra were recorded on a Hitachi S-4800 scanning electron microscope (Hitachi, Ltd., Tokyo, Japan), with ~10 μL of water-suspended nanoparticles on aluminium foil. Transmission electron microscopy (TEM), high-resolution transmission electron microscopy (HRTEM), selected area electron diffraction (SAED), and elemental mapping images were collected using a JEOL JEM-2100F instrument (JEOL Ltd., Tokyo, Japan), with ~10 μL of water-suspended nanoparticles deposited onto a copper grid with mesh diameter of 100 μm (Beijing XXBR Technology Co., Ltd, Beijing, China). Dynamic light scattering (DLS) and zeta potential measurements were conducted using a Nano-ZS90 instrument (Malvern, Worcestershire, UK) with nanoparticles dispersed in water at 25°C. Ultraviolet-visible (UV-vis) absorption and Fourier transform infrared (FTIR) spectra of the materials were obtained using a UV1900 spectrophotometer (Shimadzu Corporation, Kyoto, Japan) and a Nicolet 6700 FT-IR spectrometer (Thermo Fisher Scientific Inc., Massachusetts,USA), respectively. Digital images were taken using a Huawei Wheat 5 phone.

Cohort characteristics and serum collection

A total of 517 individuals were consecutively recruited from May 2017 to November 2017: 261 healthy controls (HCs) undergoing routine health care maintenance and 256 coronary heart disease (CHD) patients from the Shanghai Chest Hospital Affiliated to Shanghai Jiao Tong University. The inclusion and exclusion criterions are followed. For inclusion criterion, all CHD patients had a positive diagnosis by angiography and electrocardiogram, and all clinical information for each case was reviewed by two pathologists without knowledge of the clinical course of the patient. For myocardial infarction (MI) patients, 99th percentile cardiac troponin I (cTnI) levels were recorded by high-sensitivity immunoassay as previously described,[2] for which magnetic particles with capture antibodies were used to form immune complexes and the fluorescence intensity was converted into the cTnI concentrations in chemiluminescent reaction. For exclusion criterion, patients were excluded from the work if they had evidence of drugs or autoimmune syndromes. The blood was drawn at initial diagnosis without anaesthesia or surgery. Serum samples were collected based on a well-established protocolfrom the 261 HCs,[3] who had no clinical evidence of cardiovascular disease or other major disease and served as controls. Briefly, ~2 mL of blood was collected by venepuncture and centrifuged at 5100xg for 10 min. Then, the serum was transferred to a microtube and stored at -80°C. There was no significant difference in age or sex between HCs and CHD patients.

All the investigation protocols in this study were approved by the institutional ethical committees of the Shanghai Chest Hospital and the School of Biomedical Engineering, Shanghai Jiao Tong University (KS(P)1703 and KS1736). Written

informed consent was provided from all individuals participating in the study, and the use of their biological samples for analysis was approved for analysis in accordance with the Declaration of Helsinki.

Metabolic analysis by nanoparticle-assisted LDI-MS

Metabolic analyses of serum samples, metabolite standards, and prepared mixtures were performed using LDI-MS with nanoparticles as a matrix. Typically, 0.5 μL of analyte (serum sample, metabolite standard, or a prepared mixture) was mixed with 0.5 μL of deionized water-suspended nanoparticles or DHB/CHCAin a customized microarray (initially designed by Applied Biosystems, MDS SCIEX, Foster City, CA,USA) for direct LDI-MS detection. For each sample, a given number (5-50) of independent experiments were performed and the average mass spectra were extracted as serum metabolic profiles (SMPs). Specifically, LDI-MS was performed on a 5800 Proteomics Analyzer (Applied Biosystems, Framingham, MA, USA) with a Nd:YAG laser (1 kHz, 355 nm) in positive reflection mode with a repetition rate of 200 Hz and an acceleration voltage of 20 kV. The delay time for the experiments was optimized to 200 ns, and the number of laser shots per analysis was set as 200.[4] Mass calibration was carried out using standard molecules.

Machine learning for the diagnosis of CHD

The machine learning algorithms (sparse machine learning (elastic net analysis) and orthogonal projections to latent structures discriminant analysis (OPLS-DA)) were

applied to the previous collected SMPs. For sparse machine learning,[5] elastic net analysis was linearly combined with the least absolute shrinkage and selection operator (LASSO) and ridge regularization. The parameters λ_1 and λ_2 were tuned during the training process to obtain the optimized model based on its area under the curve (AUC) performance. The sparse machine learning formula that we used is as follows:

$$\widehat{\beta} = \underset{\beta}{\mathbf{argmin}}\left(\frac{||Y - X\beta||_2^2}{n} + \frac{\lambda_2}{2}||\beta||^2 + \lambda_1|\beta|_1\right)$$

where $\lambda_1 \geq 0$ and $\lambda_2 \geq 0$ are parameters controlling L1 and L2 normalization, n is the sample number, X is the extracted metabolic signal, and Y is the diagnostic label ('0' for HCs and '1' for CHD/MI/non-MI patients).

The OPLS-DA algorithm was derived from partial least square discriminant analysis (PLS-DA) algorithms to reduce model complexity through the removal of non-predictive variation in X (orthogonal to Y),[6] and thereby improve model interpretation. The OPLS-DA machine learning formula that we used is as follows:

$$X = t_p p_p^T + t_o p_o^T + E$$

$$Y = t_p q_p^T + F$$

where $p_p$is the Y-predictive loading matrix for X, $t_p$is the Y-predictive score matrix, $p_o$is the Y-orthogonal loading matrix for X, $t_o$is the Y-orthogonal score matrix, $q_p$is the Y-predictive loading matrix for Y, and E and F are the residual matrices for X and Y, respectively.

For the cross-validation, we repeated the process for 20 times with shuffled X and Y each time for the consideration of overfitting effects and the choice of best cross-validation models, where X represents the extracted serum metabolic profiles

(SMPs), and Y is the diagnostic label ('0' for HCs and '1' for CHD patients).For the permutation test, we randomly permuted diagnostic labelfor 1,000 times and calculated the distribution of AUC using the uninformative data obtained by random permutation.For valid comparison of the two algorithms, both were tested with the same methodology using Python (version 3.7).

Deep learning for enhanced MS and diagnosis

The overall architecture of the deep stabilizer, which included both a generator and discriminator, was designed based on a generative adversarial network (GAN) and trained by Wasserstein GAN (WGAN) with a gradient penalty strategy.[7]

The generator was designed with convolutional neural networks and consisted of two main branches. One branch was for stabilization-oriented spectrum reconstruction, and the other was for attention-guided peak refinement.[8] Final stabilized spectra were reconstructed as the element-wise sum of coarse-grained spectra and refined peaks.

The branch used for stabilization-oriented spectra reconstruction comprised a decoder and an encoder. A skip connection was introduced to convey information from the encoder to the decoder for fast gradient update and information reuse. The encoder was designed for feature extraction, and the decoder was designed for reconstruction, as follows:

$$\mathbf{g(x)} = \sigma(W_{enc} * F_{raw} + B_{enc})$$

$$F_{cg} = \sigma(W_{dec} * \mathbf{g(x)} + B_{dec})$$

where F_{raw} , F_{cg} , W_{enc} , W_{dec} , B_{enc} , and B_{dec} represented the input spectra, reconstructed coarse-grained spectra and convolution kernels in the encoder (enc) and decoder (dec), respectively, and the asterisk indicated the convolution operation. The leaky rectified linear unit (LeakyReLU) was chosen as our activation function, and σ was defined as follows:

$$\sigma = \max(0, x) + \alpha \min(0, x).$$

with α =0.01 to repair the "dying ReLU" problem.

To strengthen feature propagation and alleviate the vanishing gradient problem, we chose the following dense block as the basic block:[9]

$$z_l = H_l([z_0, z_1, z_2, \ldots, z_{l-1}])$$

where $[z_0, z_1, z_2, \ldots, z_{l-1}]$ represented the concatenation of previous layers' feature maps.

The branch for attention-guided peak refinement contained only one dense block with l and k set as 4 and 32, respectively:

$$F_{peaks} = \sigma(W_p * F_{raw} + B_p)$$

where F_{peaks} , F_{raw} , W_p , and B_p represented the refined peaks, input spectra, and their corresponding convolutional kernels, respectively.

For final stabilized spectrum reconstruction based on refined peaks and reconstructed coarse-grained spectra, the stabilized spectra were built by their element-wise product and element-wise sum:

$$F_{fg} = F_{cg} + F_{cg \otimes F_{peaks}}.$$

The discriminator contained 7 convolutional layers followed by 2 fully connected layers. Each convolutional layer was followed by a LeakyReLU activation layer with a negative slope of 0.2. Every convolutional layer had a stride size of 2. The loss function of the deep stabilizer consisted of two components: adversarial loss and reconstruction loss.

For adversarial loss, a variant of GAN, WGAN with gradient penalty, was adapted as the GAN framework, which solved the slow convergence and mode collapse problems. The MS reconstruction's objective function of WGAN was defined as follows:

$$\mathbb{E}_{\tilde{x}\sim\mathbf{P}_g}[D(\tilde{x})] - \mathbb{E}_{x\sim\mathbf{P}_r}[D(x)] + \lambda\mathbb{E}_{\hat{x}\sim\mathbf{P}_{\hat{x}}}[(\|\nabla_{\hat{x}}D(\hat{x})\|_2 - 1)^2]$$

where $\tilde{x}$, $\mathbf{x}$ and $\hat{x}$ represented the reconstructed spectra, high-quality spectra and interpolated spectra, respectively, and $\hat{x} = \epsilon x + (1-\epsilon)\tilde{x}$ represented the gradient penalty.

For reconstruction loss, the network was trained with a sliding window to make the deep stabilizer more robust. To decrease sensitivity to outliers, the Huber loss was selected over the mean square error (MSE) loss as the loss function. The Huber loss was defined as follows:

$$\mathcal{L}(y, \tilde{y}) = \frac{1}{n}\sum_i z_i$$

where z_i was given as follows:

$$z_i = \begin{cases} \frac{1}{2}(y_i - \tilde{y}_i)^2, & if\ |y_i - \tilde{y}_i| < 1 \\ |y_i - \tilde{y}_i| - \frac{1}{2}, & otherwise \end{cases}$$

with y_i and $\tilde{y}_i$ representing the observed value and the predicted value, respectively. Implementation was carried out in Python 3.7 with PyTorch (version 1.3.1) and torchvision (version 0.4.2).

For optimization, deep learning performance was correlated to its architecture settings. The filter number, filter size, dense block size and number of blocks were modified to study the relationships between performance and parameters. During training, the sliding-window strategy was applied to the training dataset. The Adam optimizer was used with an initial learning rate of 0.0001,[10] β_1 of 0.9 and β_2 of 0.999. Fora small subset of the training setas minibatch, 128 random sequences were used as the input. The training process was carried out on a Nvidia GeForce GTX 1080Ti GPU (Nvidia Corporation, California, USA) for 300 epochs. When testing, a whole spectrum obtained with few laser shots was used as the input to reconstruct a spectrum obtained with many laser shots.

The capability of stabilization-oriented spectrum reconstruction and attention-guided peak refinement was critical for reconstructing high-quality MS data. For attention-guided peak refinement, an ablation study was adopted, which remove single part of the model to obtain the influence on performance systematically. Specifically, the study was performed for an attention operation,while diagnostic performance was compared with and without an attention mechanism.

Statistical Analysis

Pre-processing of data

Resampling, smoothing, peak extraction, and peak alignmentwere performed in the pre-processing of MS.

Sample size (n)

The minimal sample size was decided by power analysis.Power analysis was performed by uploading 16 samples as the pilot metabolicdata into MetaboAnalyst and the predicted power for estimating the effect sample size was set as 0.8.

Statistical methods

Sensitivity, specificity, and accuracy were defined as follows:

sensitivity = TP/(TP + FN)

specificity = TN/(TN + FP)

accuracy = (TP +TN)/(TP + FP + TN + FN)

where TP was the number of true positives, FN was the number of false negatives, TN was the number of true negatives, and FP was the number of false positives.

The confidence interval was defined as follows:

$$\mathbf{CI} = \left(\bar{X} - t_{\frac{\alpha}{2}}(n-\mathbf{1})\frac{S}{\sqrt{n}}, \bar{X} + \frac{t_{\frac{\alpha}{2}}(n-\mathbf{1})S}{\sqrt{n}} \right)$$

where $\bar{X}$ represented the average value of the observed data, α represented 1-the confidence coefficient, n represented the sample number, and S represented the standard variation in the observed data.

CV was defined as follows:

$$\mathbf{CV} = \frac{\sigma}{\mu}$$

where σ represented the standard deviation of intensity or resolution and μ represented the mean intensity or resolution.

The S/N was calculated as follows:

$$\frac{\mathbf{S}}{\mathbf{N}} = \mathbf{10}\log\left(\frac{\sum_{n=1}^{N} x^2(n)}{\sum_{n=1}^{N}[x(n) - y(n)]^2}\right)$$

where y(n) and x(n) represented the spectra before and after baseline correction, respectively, and N represented the signal length.

PSNR was defined as follows:

$$\mathbf{MSE} = \frac{\sum_{i=0}^{n}(y_i - \tilde{y}_i)^2}{n}$$

$$\mathbf{PSNR} = \mathbf{10}\log_{10}\left(\frac{\max(y)^2}{MSE}\right)$$

where y represented the predicted sequence and $\tilde{y}$ represented the ground truth sequence.

Software used for statistical analysis

The chi-square test and t-test were implemented with scipy (version 1.3.3, www.scipy.org).[11]

AUPRCs and AUCs were measured with sklearn (version 0.21.3, www.scikit-learn.org).[12]

References

[1] L. Huang, L. Wang, X. Hu, S. Chen, Y. Tao, H. Su, J. Yang, W. Xu, V. Vedarethinam, S. Wu, B. Liu, X. Wan, J. Lou, Q. Wang, K. Qian, Nat. Commun. 2020, 11, 3556.

[2] a) A. S. V. Shah, A. Anand, F. E. Strachan, A. V. Ferry, K. K. Lee, A. R. Chapman, D. Sandeman, C. L. Stables, P. D. Adamson, J. P. M. Andrews, M. S. Anwar, J. Hung, A. J. Moss, R. O'Brien, C. Berry, I. Findlay, S. Walker, A. Cruickshank, A. Reid, A. Gray, P. O. Collinson, F. S. Apple, D. A. McAllister, D. Maguire, K. A. A. Fox, D. E. Newby, C. Tuck, R. Harkess, R. A. Parker, C. Keerie, C. J. Weir, N. L. Mills, S. I. High, Lancet 2018, 392, 919; b) T. Reichlin, A. Irfan, R. Twerenbold, M. Reiter, W. Hochholzer, H. Burkhalter, S. Bassetti, S. Steuer, K. Winkler, F. Peter, J. Meissner, P. Haaf, M. Potocki, B. Drexler, S. Osswald, C. Mueller, Circulation 2011, 124, 136.

[3] B. Liu, Y. Li, H. Wan, L. Wang, W. Xu, S. Zhu, Y. Liang, B. Zhang, J. Lou, H. Dai, K. Qian, Adv. Funct. Mater. 2016, 26, 7994.

[4] a) X. Sun, L. Huang, R. Zhang, W. Xu, J. Huang, D. D. Gurav, V. Vedarethinam, R. Chen, J. Lou, Q. Wang, J. Wan, K. Qian, ACS Central Sci. 2018, 4, 223; b) L. Huang, J. Wan, X. Wei, Y. Liu, J. Huang, X. Sun, R. Zhang, D. D. Gurav, V. Vedarethinam, Y. Li, R. Chen, K. Qian, Nat. Commun. 2017, 8, 220.

[5] J. Friedman, T. Hastie, R. Tibshirani, J. Stat. Softw. 2010, 33, 1.

[6] J. Trygg, S. Wold, J. Chemometr. 2002, 16, 119.

[7] a) I. J. Goodfellow, J. Pouget-Abadie, M. Mirza, B. Xu, D. Warde-Farley, S. Ozair, A. Courville, Y. Bengio, in *Advances in Neural Information Processing Systems 27*, Vol. 27 (Eds: Z. Ghahramani, M. Welling, C. Cortes, N. D. Lawrence, K. Q. Weinberger), 2014; b) M. Arjovsky, S. Chintala, L. Bottou, in *Proceedings of the 34th International Conference on Machine Learning*, Vol. 70 (Eds: P. Doina, T. Yee Whye), PMLR, Proceedings of Machine Learning Research 2017, 214.

[8] A. Vaswani, N. Shazeer, N. Parmar, J. Uszkoreit, L. Jones, A. N. Gomez, L. Kaiser, I. Polosukhin, in *Advances in Neural Information Processing Systems 30*, Vol. 30 (Eds: I. Guyon, U. V. Luxburg, S. Bengio, H. Wallach, R. Fergus, S. Vishwanathan, R. Garnett), 2017.

[9] G. Huang, Z. Liu, L. van der Maaten, K. Q. Weinberger, in *30th IEEE Conference on Computer Vision and Pattern Recognition*, 2017, 2261.

[10] D. Kingma, J. Ba, in *International Conference on Learning Representations*, 2014.

[11] Y. Zhao, R. L. Antoniou-Kourounioti, G. Calder, C. Dean, M. Howard, Nature 2020, 583, 825.

[12] F. Pedregosa, G. Varoquaux, A. Gramfort, V. Michel, B. Thirion, O. Grisel, M. Blondel, P. Prettenhofer, R. Weiss, V. Dubourg, J. Vanderplas, A. Passos, D. Cournapeau, M. Brucher, M. Perrot, E. Duchesnay, J. Mach. Learn. Res. 2011, 12, 2825.

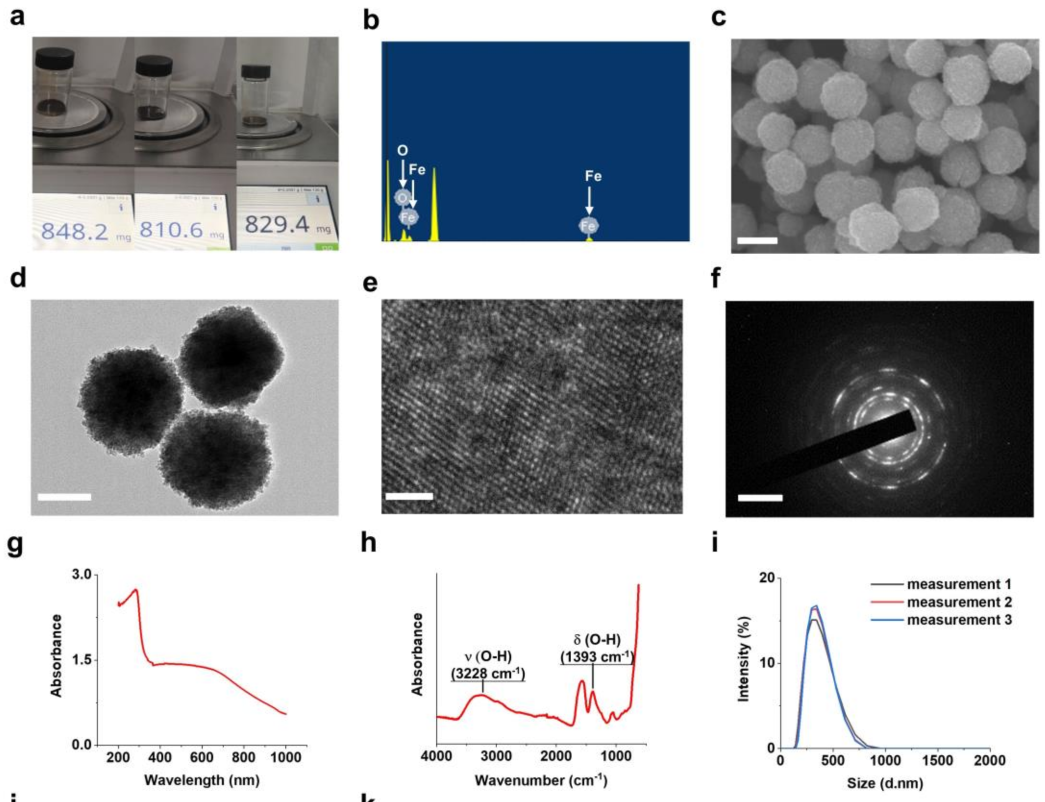

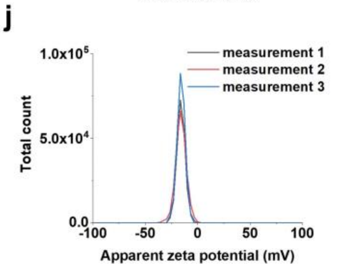

k

Sample	Size (nm)	PDI	Zeta potential (mV)	Weight (%)		Atomic (%)	
				Fe	O	Fe	O
Ferrous nanoparticles	325.8 ± 6.2	0.166 ± 0.11	16 ± 0.2	64.11	35.89	33.85	66.15

Figure S1. Physicochemical properties of ferrous nanoparticles. (a) Digital images of the products of three independent rounds of synthesis, showing that synthesis yielded ~0.83 g of ferrous nanoparticles per batch. (b) Typicalenergy-dispersive X-ray (EDX)spectrum and (c) scanning electron microscopy (SEM)image of ferrous nanoparticles showing nanoscale surface roughness. (d-f) Transmission electron microscopy (TEM), (d) high-resolution TEM (e) andselected area diffraction (SAED) (f) imagesof the material.The scale bars were 200 nm in (c), 100 nm in (d), 2 nm in (e), and 5 1/nm in (f). (g) Ultraviolet-visible (UV-vis) spectrum of ferrous nanoparticles. (h) Fourier transform infrared (FTIR) spectrum of ferrous nanoparticles showing the main bands at 3228 and 1393 cm^{-1}, which were assigned to O-H stretching and O-H bending, respectively. (i) Size distribution of ferrous nanoparticles, as determined using

dynamic light scattering (DLS) analysis. (j) Zeta potential distribution of ferrous nanoparticles. (k) Summary of the structural parameters of the ferrous nanoparticles. Three independent experiments were performed to determine both size and zeta potential distribution.

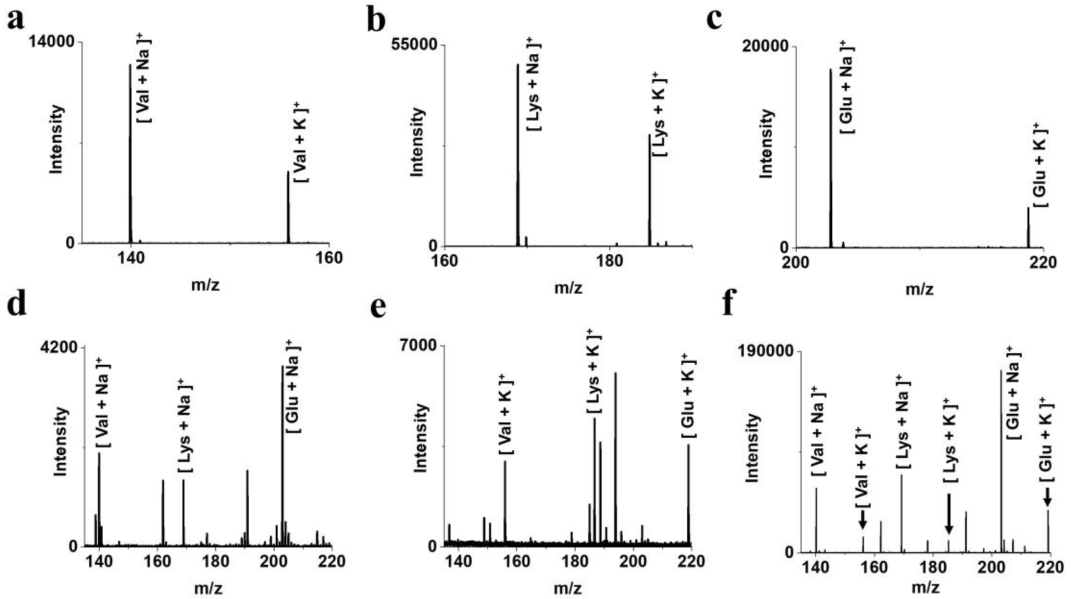

Figure S2. Nanoparticle-assisted LDI-MS detection of small metabolite standards. Typical results of LDI-MS detection of 0.5 μL of mixtures containingthe following small metabolite standards using ferrous nanoparticles: (a) valine (Val, 1 ng/nL), (b) lysine (Lys, 1 ng/nL), and (c) glucose (Glu, 1 ng/nL). A mixture of Val, Lys, and Glu (each at 1 ng/nL) in a (d) NaCl solution (0.5 M) and a (e) KCl solution (0.5 M) was analysed. A mixture of Val, Lys, and Glu (each at 1 ng/nL) in a (f) bovine serum albumin (BSA, 5 mg/mL) solution was analysed. All the results were recorded in positive ion mode, and Na^+/K^+ adducts were observed.

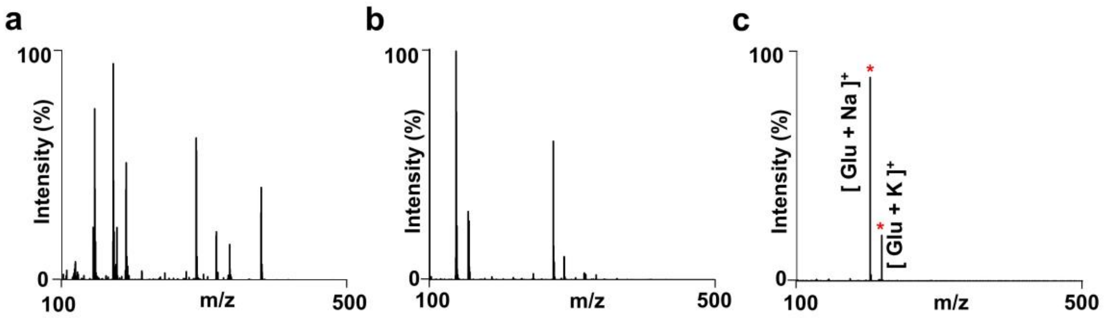

Figure S3. LDI MS of glucose using different matrices. LDI mass spectra of 0.5 μL of glucose (Glu, 1 ng/nL) using (a) CHCA, (b) DHB, and (c) ferrous nanoparticles in positive mode.

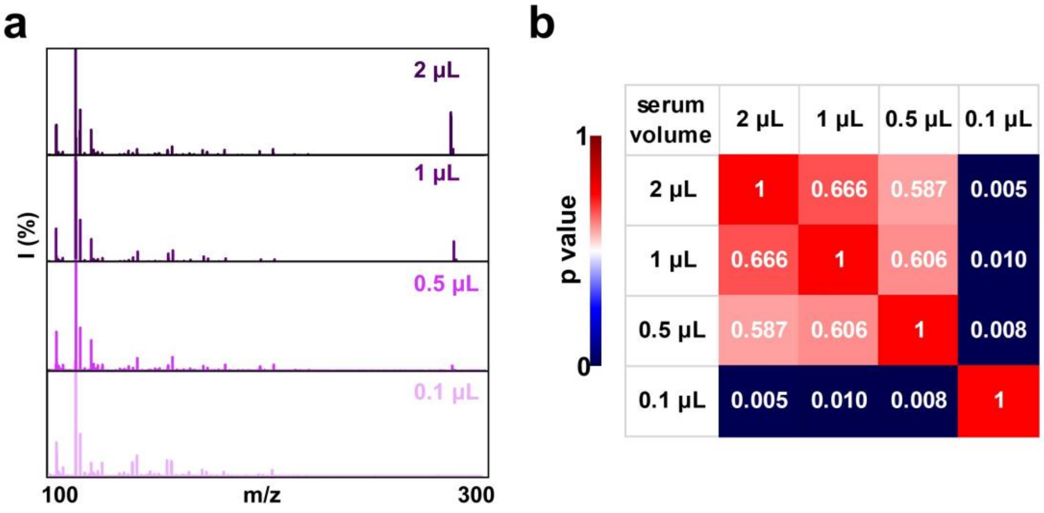

Figure S4. Optimization of serum volume in nanoparticle-assisted LDI-MS detection. Optimization of serum volume was carried out by nanoparticle-assisted LDI using 2 μL, 1 μL, 0.5 μL, and 0.1 μL serum**.** (a) Typical MS spectra within a m/z range from 100 to 300 obtained by nanoparticle-assisted LDI using 2 μL, 1 μL, 0.5 μL, and 0.1 μL serum samples. (b) The statistical analysis of detected peak numbers using 2 μL, 1 μL, 0.5 μL, and 0.1 μLserum with five independent measurements by independent-samples t-test.

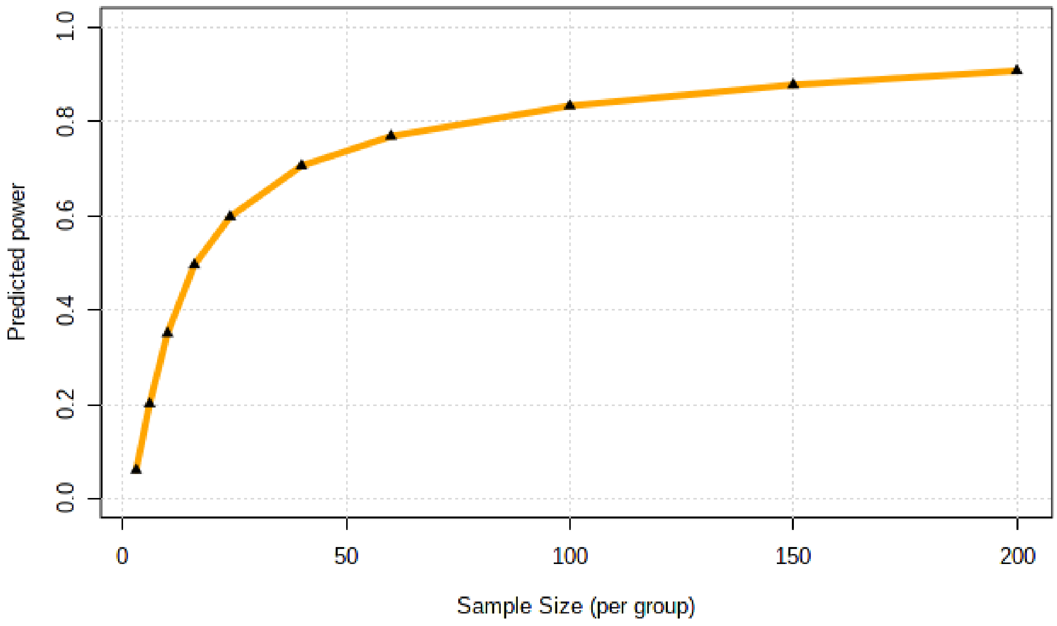

Figure S5. Power analysis of pilot study. The serum metabolic profiles (SMPs) extracted from 16 samples (8/8, CHD/control) were analyzed to obtain the minimum number of samples required with meaningful machine learning. Minimum sample size of 80 for each group will achieve the predicted power of>0.8, which is significant to conclude the statistical meaningful results.

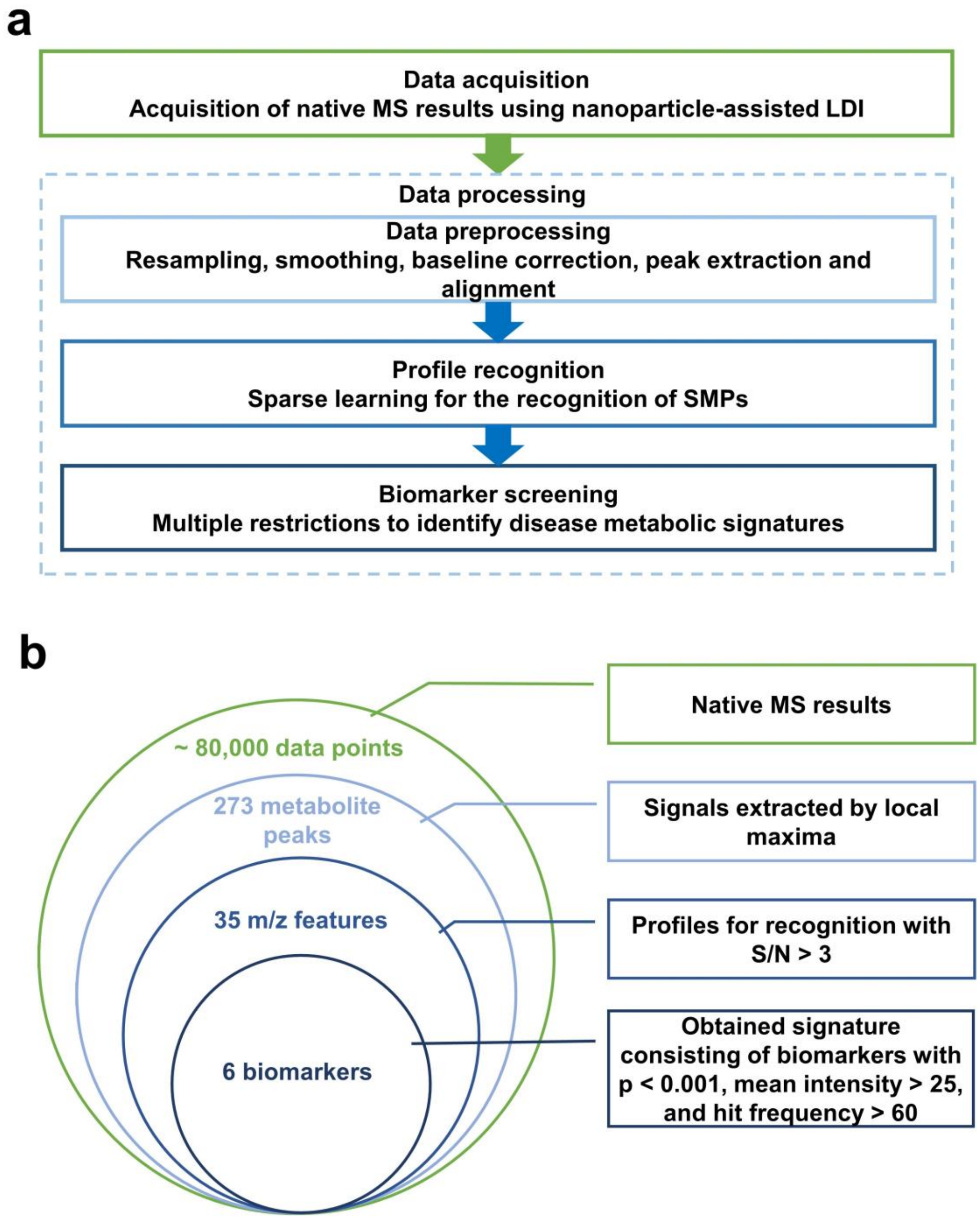

Figure S6. Procedures for data acquisition and processing and Venn diagram showing the obtained data.(a) After data acquisition using nanoparticle-assisted LDI-MS, the following data processing procedures were carried out: signal extraction by preprocessing, feature selection by the determination of signal-to-noise (S/N) ratios, profile recognition by sparse machine learning, and biomarker screening by multiple restrictions. (b) Venn diagram showing the numbers of native MS data points, metabolite peaks, m/z features and biomarkers in the obtained signature.

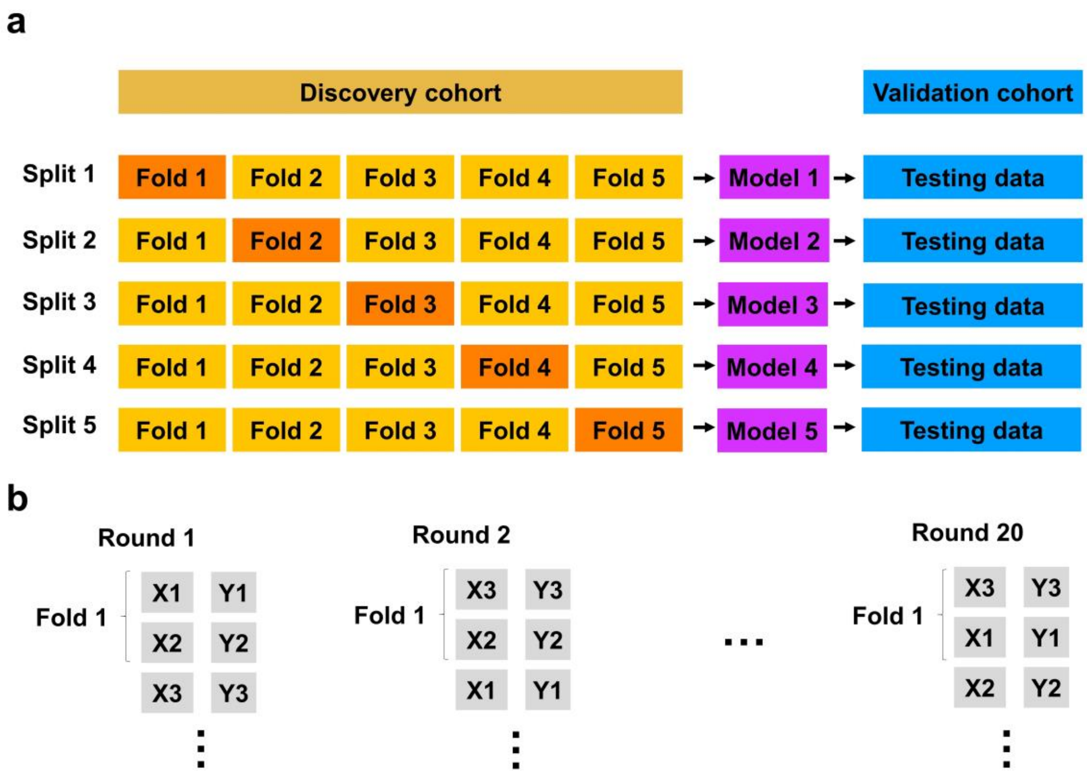

Figure S7. Diagnostic model construction with cross-validation. (a) 5-fold cross-validation based on discovery cohort. Four folds were used for training, one was used for validation, and validation cohort was used for testing. (b) Work flow of shuffled X and Y (20 times). X1/X2/X3 and Y1/Y2/Y3 represented the SMPs and labels for sample 1/2/3, respectively.

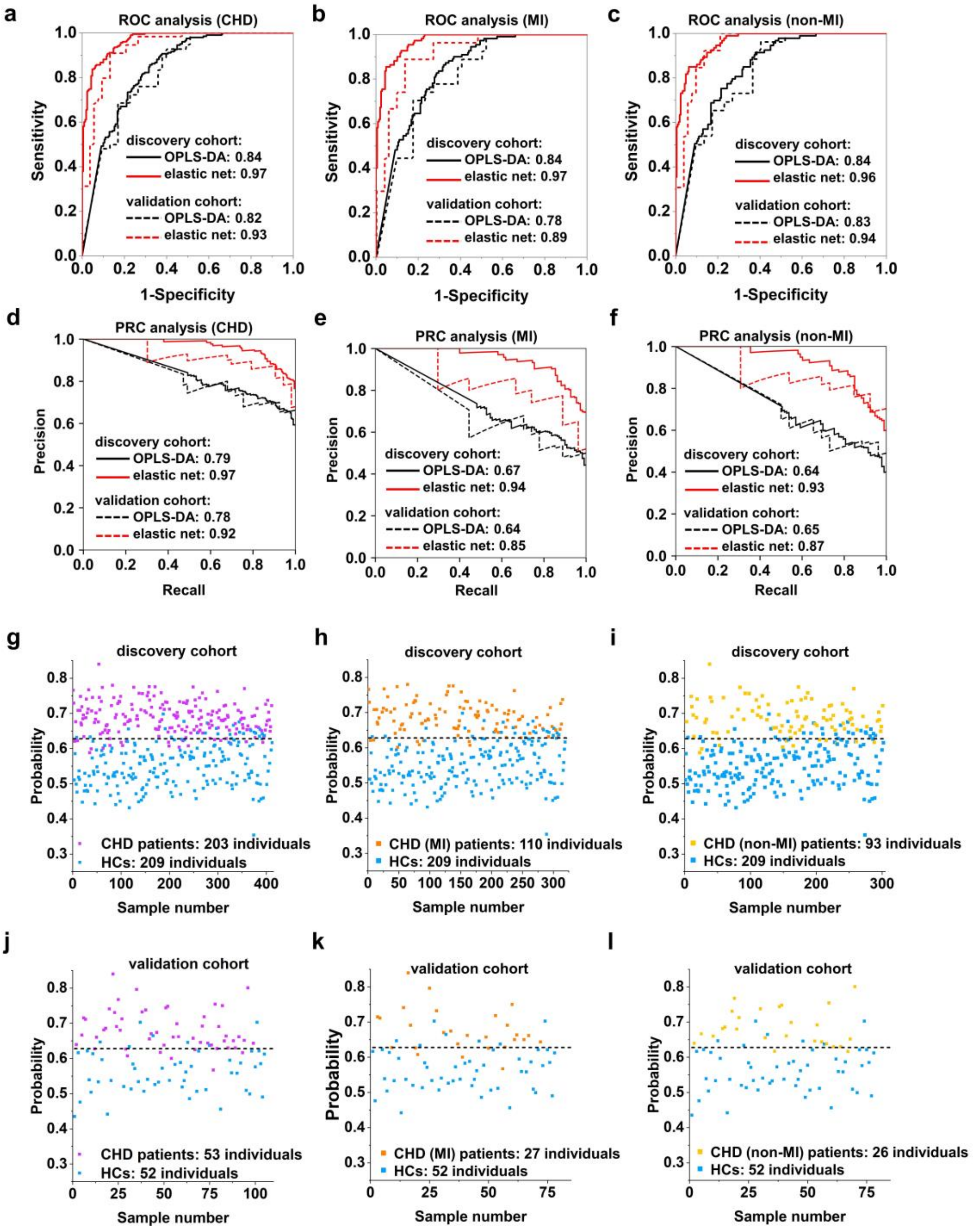

Figure S8. Diagnostic performance using machine learning for stratification and prediction of MI and non-MI CHD patients against HCs. (a-c) Receiver operating characteristic (ROC) curves and areas under the curve (AUC) obtained using orthogonal projections to latent structures discriminant analysis (OPLS-DA, in black) and sparse learning (elastic net analysis, in red) to distinguish (a) CHD, (b) MI, and (c) non-MI patients from HCs. The solid and dashed lines represented the results from the

discovery and validation cohorts, respectively. (d-f) Precision-recall curves (PRC) analysis using orthogonal projections to latent structures discriminant analysis (OPLS-DA, black) and sparse learning (elastic net analysis, red) to distinguish HCs from (d) CHD, (e) MI, and (f) non-MI patients. The solid and dashed lines showed the results from the discovery and validation cohorts, respectively.Stratification based on the predicted probability of CHD/MI/non-MI patients and HCs by sparse learning (elastic net analysis) with serum metabolic profiles (SMPs) in the (g-i) discovery cohort and (j-l) validation cohort. The dashed lines in (g-l) indicated the machine-learning-derived threshold to distinguish between MI patients and HCs.The blue and purple/orange/yellow points represented HCs and CHD/MI/non-MI patients, respectively.

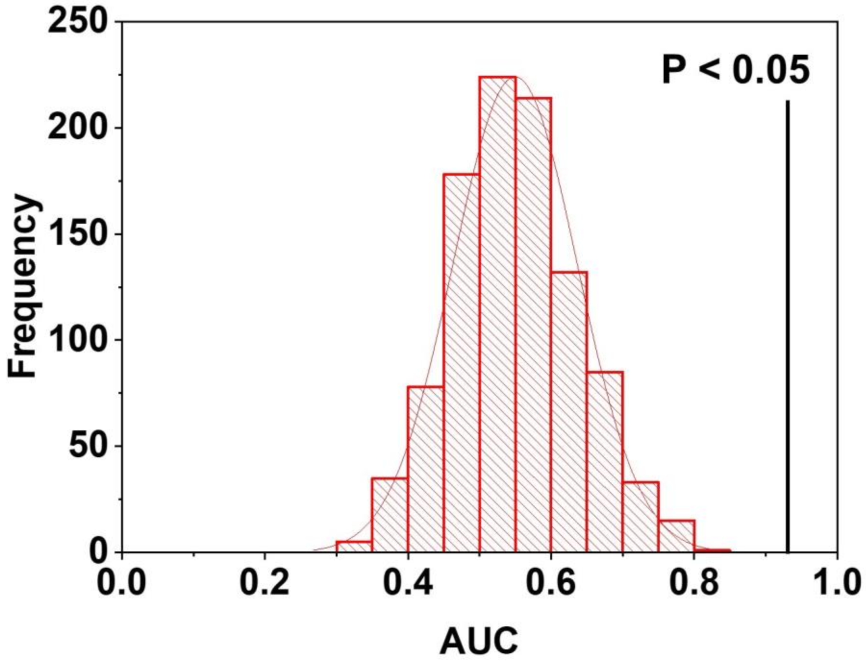

Figure S9. Permutation test of classifier. Distribution of AUC calculated using the uninformative data obtained by random permutation (1,000 permutations).

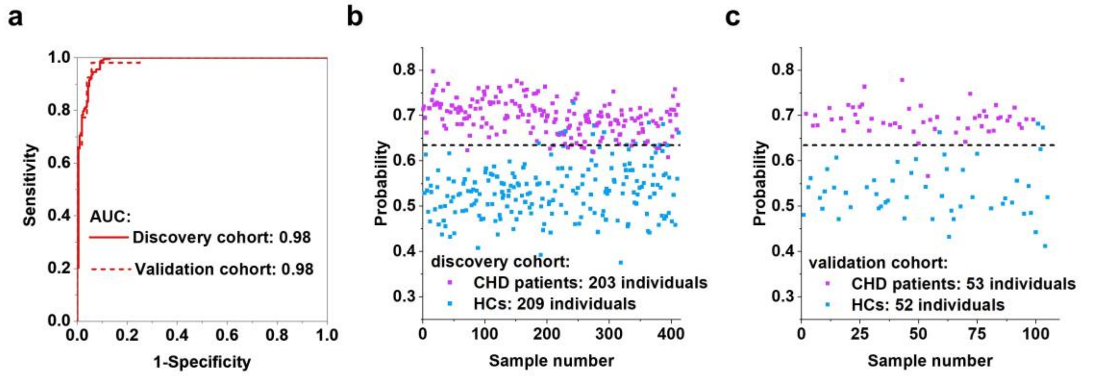

Figure S10. Diagnostic performance using machine learning for stratification and prediction with reference MS spectra.Reference MS spectra were obtained using a large number of tests. (a) Receiver operating characteristic (ROC) curves and areas under the curve (AUCs) obtained using sparse learning (elastic net analysis) to distinguish HCs from CHD patients. The solid and dashed lines represented the results from the discovery and validation cohorts, respectively. Stratification was based on the predicted probability of CHD patients and HCs by sparse learning (elastic net analysis) of SMPs in the (b) discovery cohort and (c) validation cohort. The blue and purple points represented HCs and CHD patients, respectively. The dashed lines in (b-c) indicated the machine-learning-derived threshold to distinguish CHD patients from HCs.

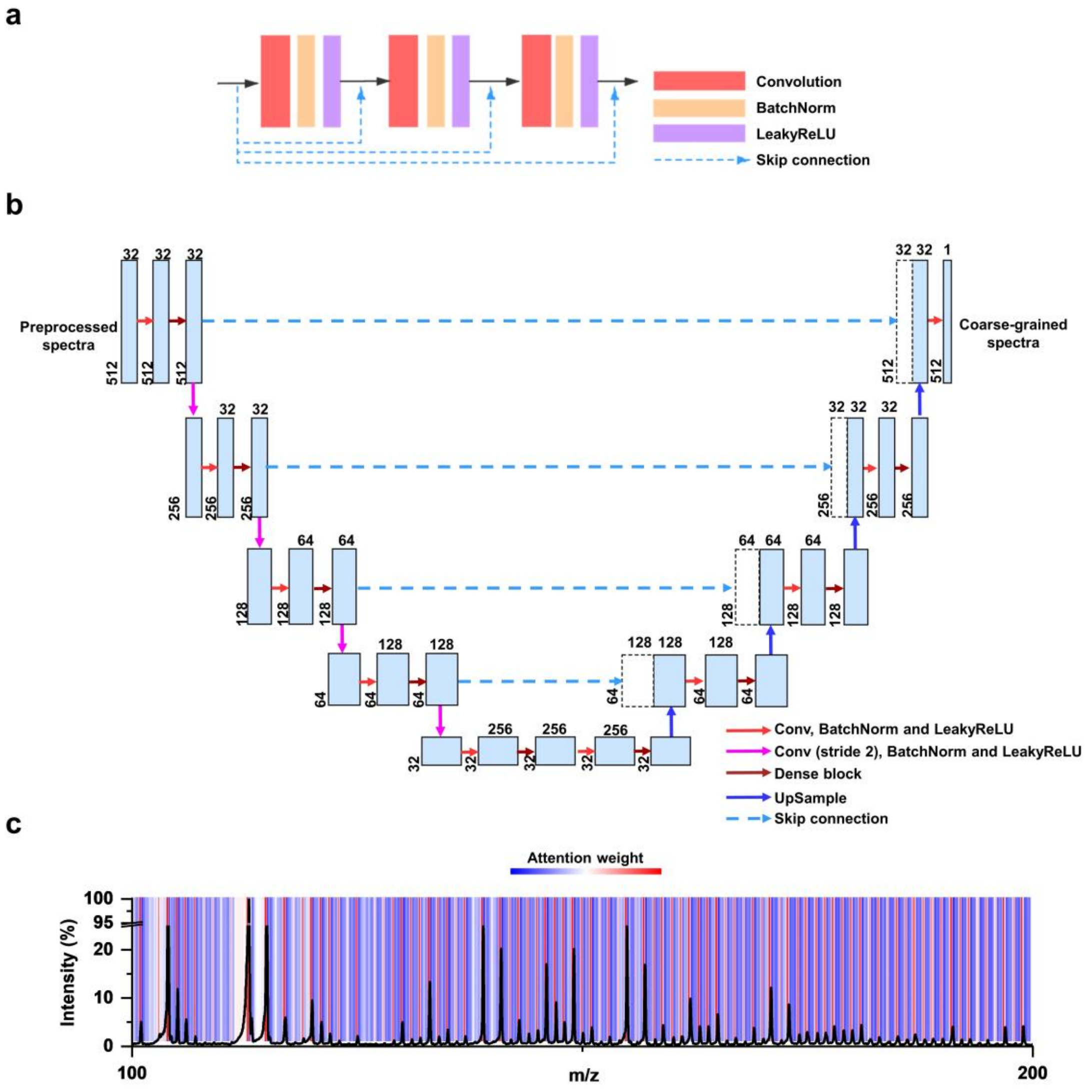

Figure S11. Detailed network architecture for the deep stabilizer.(a)Dense block structure.Each layer uses all preceding feature maps as input, and features were combined through concatenation. There were n inputs in the nth layer, which consisted of the feature maps of all preceding convolutional blocks. The features in the nth layer were passed to all subsequentlayers. (b)Network structure for the reconstruction of stabilization-oriented spectra. Each blue box referred to a multi-channel feature map. The number of channels was annotated on the top of each box. The number of training spectra (used in a sliding-window manner) was denoted at the lower left edge of each box. The white box corresponded to the copied feature maps. The arrows represented different operations that were described lower right corner of the network structure.(c) Demonstration of attention-guided peak refinement. The spectrum referred to stabilized

MS data. The heatmap in the background indicated the attention weight for peaks, which was calculated by applying a dense block to the pre-convolved spectrum and activated using a sigmoid function.

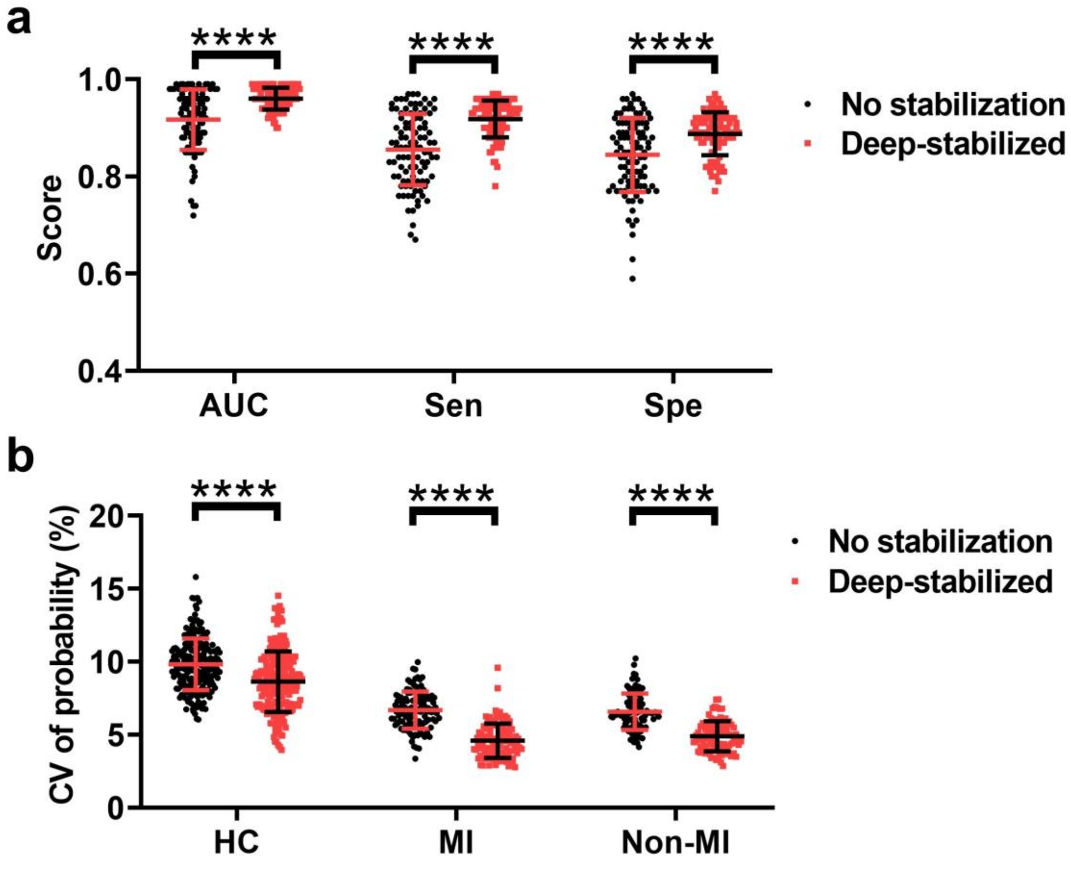

Figure S12. Diagnostic performance of the deep stabilizer in the discovery cohort.(a) Diagnostic performance (AUC, sensitivity (Sen), and specificity (Spe)) for CHD via no stabilization and deep-stabilized experiments in the discovery cohort. (b) CVs of predicted probabilities for HCs and CHD (MI and non-MI) patients via no stabilization and deep-stabilized experiments in the discovery cohort. **** indicated $p < 0.0001$ in independent-samples t-test.

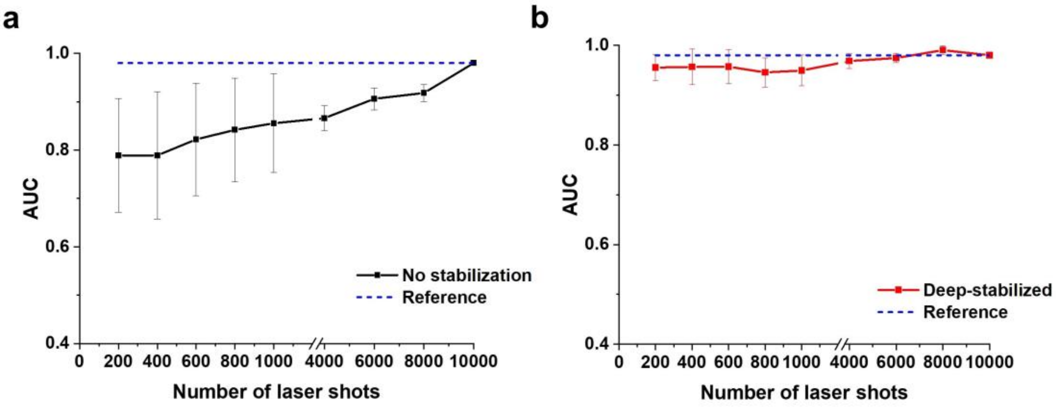

Figure S13. Diagnostic performance with different numbers of laser shots.The number of shots used to obtain the MS data used as input for the deep stabilizer was varied, and the resulting diagnostic performance in distinguishing CHD patients from HCs with (a) no stabilizationMS data and (b) deep-stabilized MS data was determined. The dashed blue line indicated the diagnostic performance with the reference MS data. The black solid line indicated the diagnostic performance with the no stabilizationMS data. The red solid line indicated the diagnostic performance with the deep-stabilized MS data. The error bars indicated the AUC score (mean ± SD).

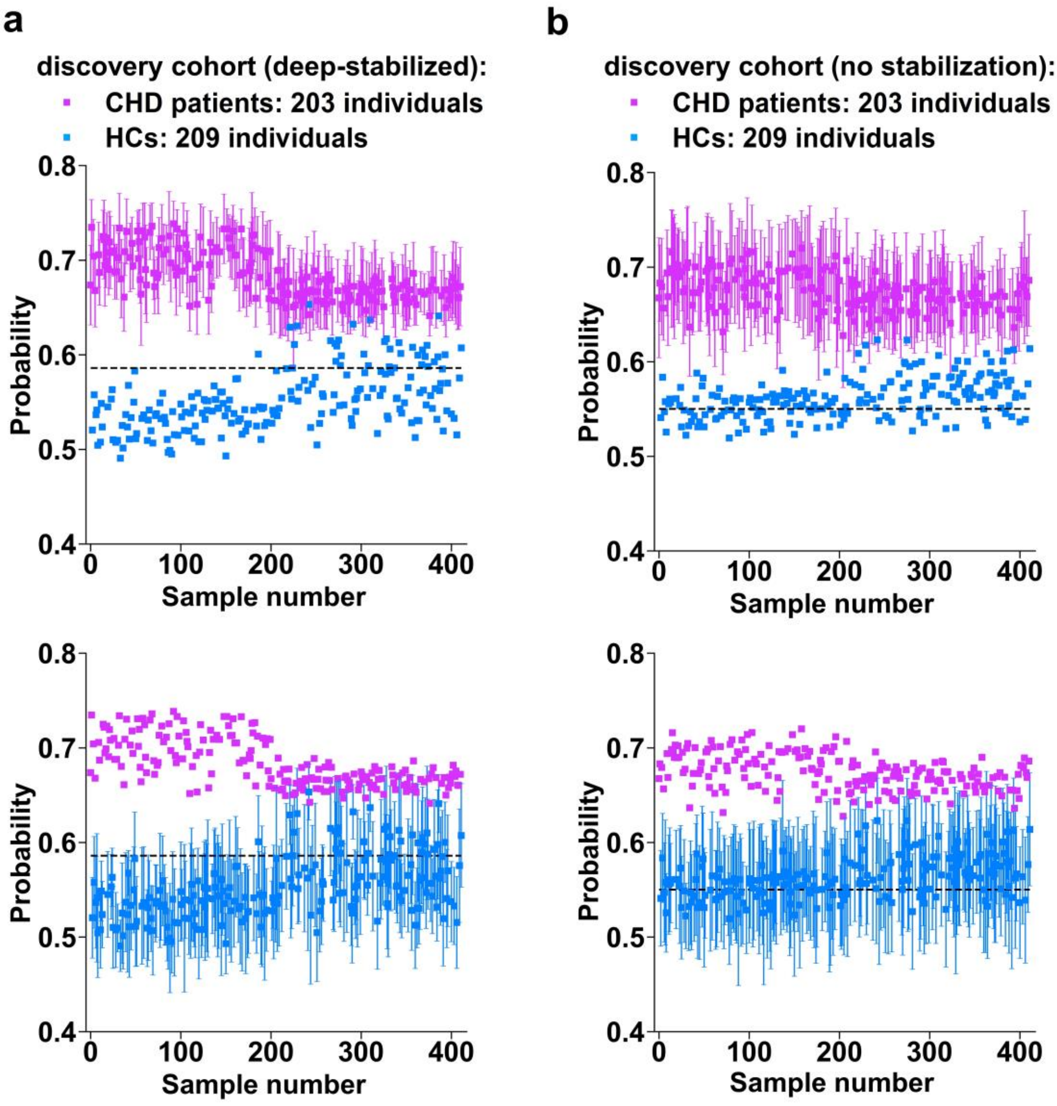

Figure S14. Stratification results for CHD patients and HCs in the discovery cohort by machine learning in 100 inter-replication combinations. Stratification of the predicted probabilities of CHD patients and HCs by elastic net analysis of SMPs determined from (a) deep-stabilized MS and (b) no stabilizationMS results. Blue and purple points represented the means of predicted probabilities of HCs and CHD patients, respectively. Dashed lines indicated the threshold derived from a CV of 10% to distinguish between CHD patients and HCs. The errors bars indicated the standard deviation of the predicted probability of HCs (lower) and CHD patients (upper).

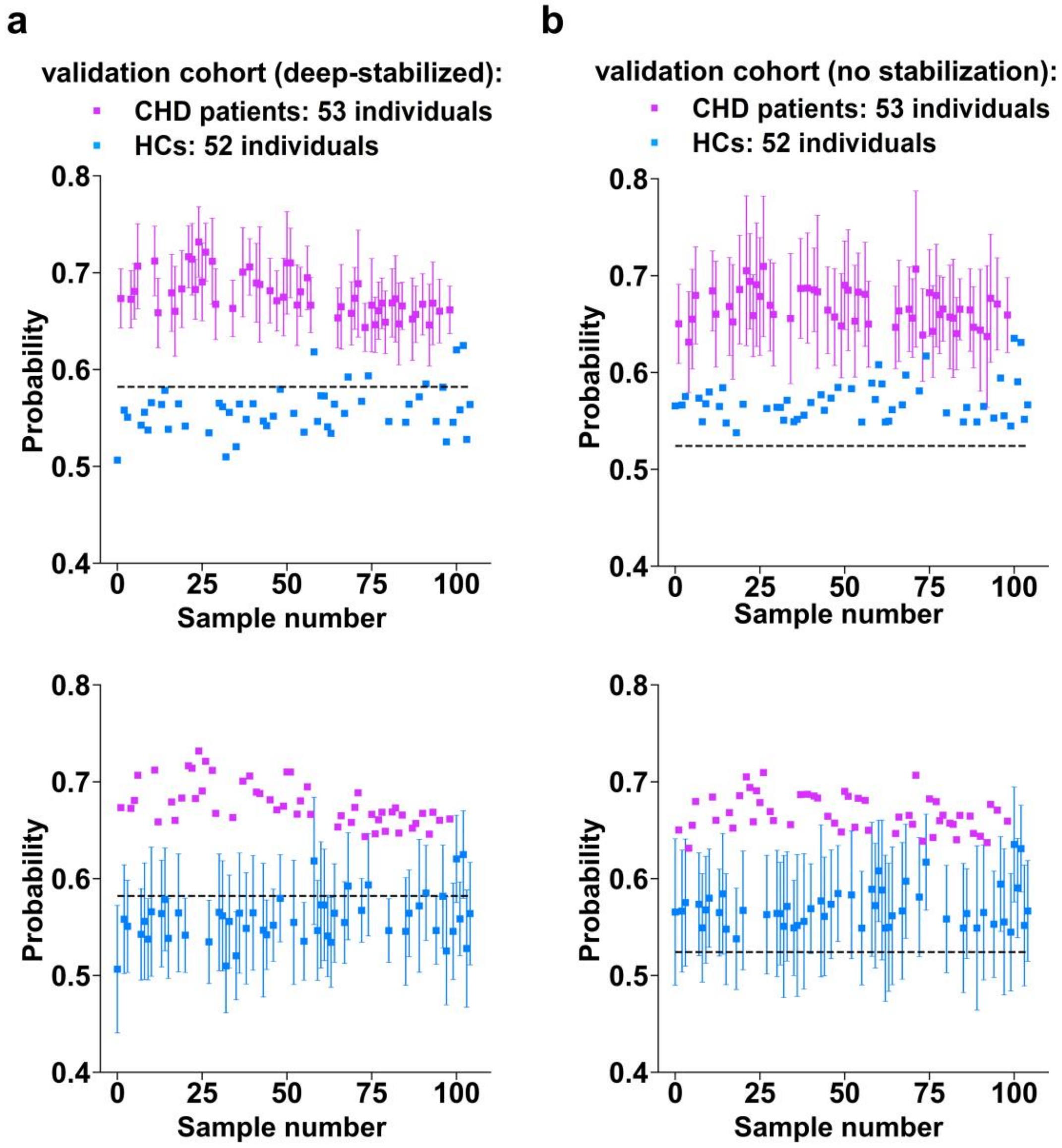

Figure S15. Stratification of CHD patients and HCs in the validation cohort determined by machine learning in 100 inter-replication combinations. Stratification of the predicted probabilities of CHD patients and HCs determined by elastic net analysis of SMPs from (a) deep-stabilized MS and (b) no stabilizationMS spectra. Blue and purple points represented the mean of the predicted probability of HCs and CHD patients, respectively. Dashed lines indicated the threshold to distinguish between CHD patients and HCs derived from a CV of 10%. The error bars indicated the standard deviations of the predicted probabilities of HCs (lower) and CHD patients (upper).

Table S1. Detection limits for typical metabolites.

Analyte[a]	MW[b] (Da)	Detection limit[c] (pmol)
Glu	180.16	55.5
Pro	115.13	86.9
Leu	131.17	76.2
Val	117.15	8.5
Lys	146.19	0.7
Tyr	181.19	552
Ala	89.09	11.2
Arg	174.20	574
Ser	105.09	1.0
Met	149.21	67

[a]Glu: glucose; Pro: proline; Leu: leucine; Val: valine; Lys: lysine; Tyr: tyrosine; Ala: alanine; Arg: arginine; Ser: serine; Met: methionine.

[b]MW referred to the molecular weights of the metabolites.

[c]The detection limit was determined by nanoparticle-assisted LDI-MS.

Table S2. Comparison of NMR spectroscopy, LC-MS, and LDI-MS for the detection of metabolites.

Method[a]	Sample volume[b] (μL)	Sample pretreatment time[c] (per batch)	Sample-in to data-out[d] time (per sample)	References
NMR	200-500	1-1.5 h	12-30 min	[1, 2, 3][e]
LC-MS	20-400	1-7 h	0.25-1.5 h	[4, 5, 6][f]
GC-MS	200-400	5.25-8 h	0.25-0.5 h	[7, 8, 9][g]
LDI-MS	0.5[h]	30 s[h]	20 s[h]	This work

[a]NMR: nuclear magnetic resonance spectroscopy; LC-MS: liquid chromatography-mass spectrometry; LDI-MS: laser desorption/ionization mass spectrometry.

[b]Sample referred to biofluids, such as serum, plasma, and urine.

[c]Sample pretreatment referred to centrifugation, chromatography, and derivatization.

[d]Sample-in to data-out time times referred to the total time for NMR, LC-MS, GC-MS, and LDI-MS analyses per sample.

[e]Typical references included:

1 Liu, P., Cooks, R. G. & Chen, H. Nuclear Magnetic Resonance Structure Elucidation of Peptide b(2) Ions. *Angew. Chem.-Int. Edit.*54, 1547-1550 (2015).

2 Wishart, D. S. Quantitative metabolomics using NMR. *Trac-Trends Anal. Chem.*27, 228-237 (2008).

3 Beckonert, O. et al. Metabolic profiling, metabolomic and metabonomic procedures for NMR spectroscopy of urine, plasma, serum and tissue extracts. *Nat. Protoc.* 2, 2692-2703 (2007).

[f]Typical references included:

4 Want, E. J. et al. Global metabolic profiling procedures for urine using UPLC-MS. *Nat. Protoc.*5, 1005-1018 (2010).

5 Rabbani, N. & Thornalley, P. J. Measurement of methylglyoxal by stable isotopic dilution analysis LC-MS/MS with corroborative prediction in physiological samples. *Nat. Protoc.*9, 1969-1979 (2014).

6 Li, H. et al. Particulate matter exposure and stress hormone levels: A randomized, double-blind, crossover trial of air purification. *Circulation*136, 618-627 (2017).

[g]Typical references included:

7 Smart, K. F., Aggio, R. B. M., Van Houtte, J. R. & Villas-Bôas, S. G. Analytical platform for metabolome analysis of microbial cells using methyl chloroformate derivatization followed by gas chromatography–mass spectrometry. *Nat. Protoc.*5, 1709-1729 (2010).

8 Dunn, W. B. et al. Procedures for large-scale metabolic profiling of serum and plasma using gas chromatography and liquid chromatography coupled to mass spectrometry. *Nat. Protoc.*6, 1060-1083 (2011).

9 Chan, E. C. Y., Pasikanti, K. K. & Nicholson, J. K. Global urinary metabolic profiling procedures using gas chromatography–mass spectrometry. *Nat. Protoc.*6, 1483-1499 (2011).

[h]Based on this work.

Table S3. Demographic information for the coronary heart disease patients and healthy controls.

Category[a]	Age (median)	Age (range)	Sex (n)		cTnI level (ng/mL)	P_1[b]	P_2[c]
			Male	Female			
HC	66	34-90	182	79	0-0.03	-	-
CHD	66	41-94	172	84	0-85	0.99	0.60
MI	67	41-94	97	40	0.04-85	0.81	0.92
non-MI	66	48-91	75	44	0-0.03	0.80	0.24

[a]Patient category included coronary heart disease (CHD) patients (denoted CHD), CHD patients with myocardial infarction (denoted MI), and CHD patients without myocardial infarction (denoted non-MI).

[b]P_1 referred to the p values for age, which were calculated by independent-samples t-test of data from patients (CHD/MI/non-MI) and HCs.

[c]P_2 referred to the p values for sex, which were calculated by χ^2 test of data from patients (CHD/MI/non-MI) and HCs.

Table S4. Screened biomarkers from the serum metabolic profile for the differentiation between coronary heart disease (CHD) patients and controls.

Metabolite[a]	HMDB ID	AUC[b]	Fre[c]	P-value[d]	Int[e]	CHD diff [f]
1-Methylpyrrole	HMDB0033113	0.60	82.00	1.04E-04	2278	↑
Glycerol	HMDB0000131	0.66	66.00	3.07E-15	2029	↑
Imidazoleacetic acid	HMDB0002024	0.62	69.00	4.09E-08	520	↑
D-2-Hydroxyglutaric acid	HMDB0000606	0.62	92.00	3.53E-08	287	↑
D-Ribose	HMDB0000283	0.65	84.00	1.30E-12	325	↑
Pyridoxamine	HMDB0001431	0.65	100.00	7.14E-07	226	↓

[a]Metabolitereferred the name of metabolites for screened biomarkers identified by human metabolome database (HMDB, http://www.hmdb.ca/).

[b]AUC referred to the area under the curve, which was calculated using the single m/z feature for diagnosis.

[c]Fre referred to the hit frequency of a single m/z feature, which was selected from 100 sparse learning models as a screened biomarker.

[d]p value referred to the p value obtained from a t-test of a single m/z feature between CHD patients and controls.

[e]Int referred to the mean intensity at a single m/z feature

[f]CHD diff referredto whether the single m/z feature was increased or decreased in intensity, in CHD patients compared to controls.

Table S5. Summary of diagnostic performance by sparse learning (elastic net analysis) and OPLS-DA.

Patient category[a]	Algorithm[b]	Discovery cohort[c]				Validation cohort[c]			
		AUC (95% CI)	Sen	Spe	Acc	AUC (95% CI)	Sen	Spe	Acc
CHD	Elastic net	0.97 (0.95-0.98)	0.91	0.88	0.89	0.93 (0.88-0.98)	0.85	0.87	0.86
	OPLS-DA	0.84 (0.80-0.88)	0.73	0.78	0.76	0.82 (0.74-0.90)	0.77	0.68	0.72
MI	Elastic net	0.97 (0.95-0.99)	0.93	0.88	0.90	0.92 (0.85-0.99)	0.89	0.87	0.84
	OPLS-DA	0.84 (0.79-0.87)	0.80	0.72	0.75	0.81 (0.71-0.92)	0.78	0.67	0.71
non-MI	Elastic net	0.96 (0.94-0.98)	0.88	0.88	0.88	0.94 (0.88-1.00)	0.92	0.87	0.88
	OPLS-DA	0.84 (0.79-0.89)	0.75	0.78	0.77	0.83 (0.72-0.93)	0.65	0.77	0.74

[a]Patients included coronary heart disease (CHD) patients (denoted CHD), CHD patients with myocardial infarction (denoted MI), and CHD patients without myocardial infarction (denoted non-MI).

[b]Algorithms included elastic net analysis and OPLS-DA, which were both tested in the same environment.

[c]Discovery/validation cohort referred to the diagnostic performance in the discovery/validation cohorts, as assessed by the area under the curve (AUC), sensitivity (Sen), specificity (Spe), and accuracy (Acc).

Table S6. Summary of cross-validation in diagnostic model by elastic net.

Round	Cross-validation AUC [a]	Testing AUC [b]	P-value [c]
1	0.9342 ± 0.0223	0.9091 ± 0.0098	0.0731
2	0.9315 ± 0.0240	0.9088 ± 0.0125	0.1316
3	0.9219 ± 0.0348	0.9126 ± 0.0142	0.6340
4	0.9228 ± 0.0192	0.9102 ± 0.0127	0.3046
5	0.9302 ± 0.0218	0.9123 ± 0.0141	0.2060
6	0.9354 ± 0.0273	0.9112 ± 0.0073	0.1249
7	0.9118 ± 0.0466	0.9083 ± 0.0077	0.8882
8	0.9189 ± 0.0217	0.9101 ± 0.0125	0.5020
9	0.9178 ± 0.0310	0.9118 ± 0.0049	0.7203
10	0.9360 ± 0.0326	0.9097 ± 0.0114	0.1662
11	0.9165 ± 0.0296	0.9115 ± 0.0073	0.7532
12	0.9102 ± 0.0389	0.9094 ± 0.0025	0.9702
13	0.9222 ± 0.0351	0.9134 ± 0.0069	0.6382
14	0.9276 ± 0.0224	0.9102 ± 0.0061	0.1716
15	0.9256 ± 0.0368	0.9108 ± 0.0145	0.4758
16	0.9178 ± 0.0250	0.9102 ± 0.0107	0.5927
17	0.9242 ± 0.0387	0.9128 ± 0.0079	0.5817
18	0.9302 ± 0.0488	0.9138 ± 0.0045	0.5216
19	0.9322 ± 0.0253	0.9100 ± 0.0067	0.1293
20	0.9286 ± 0.0367	0.9119 ± 0.0071	0.3981

[a]Cross-validation AUC referred to the AUC of validation fold in discovery cohort, represented by mean ± SD.

[b]Testing AUC referred to the AUC of validation cohort (independent blind test dataset), represented by mean ± SD.

[c]P-value was calculated between validation AUC and testing AUC by independent-samples t-test.

Table S7. Diagnostic performance (no stabilization)for the validation cohort with 100 inter-replication combinations.

No.[a]	CHD[b]			MI[b]			non-MI[b]		
	AUC	Sen	Spe	AUC	Sen	Spe	AUC	Sen	Spe
1	0.95	0.91	0.87	0.94	0.96	0.81	0.96	0.92	0.9
2	0.93	0.91	0.87	0.92	0.89	0.87	0.94	0.92	0.87
3	0.78	0.77	0.69	0.77	0.78	0.69	0.79	0.85	0.65
4	0.94	0.92	0.85	0.94	0.89	0.85	0.95	0.92	0.87
5	0.95	0.92	0.88	0.93	0.85	0.88	0.97	1	0.88
6	0.84	0.79	0.81	0.81	0.74	0.79	0.87	0.88	0.81
7	0.85	0.77	0.77	0.84	0.85	0.69	0.86	0.88	0.75
8	0.75	0.79	0.63	0.73	0.7	0.67	0.77	0.65	0.81
9	0.95	0.92	0.92	0.94	0.85	0.94	0.96	0.96	0.94
10	0.92	0.92	0.85	0.9	0.85	0.85	0.94	0.92	0.9
11	0.93	0.85	0.88	0.92	0.89	0.79	0.94	0.92	0.88
12	0.9	0.83	0.88	0.89	0.78	0.9	0.92	0.88	0.88
13	0.84	0.68	0.88	0.84	0.67	0.88	0.85	0.81	0.75
14	0.94	0.91	0.85	0.93	0.85	0.85	0.96	0.88	0.9
15	0.92	0.92	0.81	0.91	0.89	0.81	0.93	0.92	0.83
16	0.78	0.72	0.79	0.78	0.74	0.73	0.78	0.77	0.79
17	0.78	0.75	0.71	0.73	0.67	0.69	0.84	0.88	0.75
18	0.75	0.58	0.83	0.71	0.56	0.73	0.8	0.69	0.83
19	0.93	0.83	0.88	0.92	0.74	0.9	0.95	0.92	0.88
20	0.88	0.83	0.87	0.86	0.85	0.75	0.91	0.96	0.87
21	0.97	0.98	0.9	0.97	1	0.9	0.97	0.92	0.96
22	0.96	0.89	0.92	0.95	0.89	0.92	0.97	0.88	0.92
23	0.9	0.74	0.92	0.89	0.85	0.73	0.91	0.81	0.92
24	0.98	0.92	0.94	0.97	0.93	0.94	0.99	0.96	0.92
25	0.97	0.94	0.88	0.97	0.93	0.87	0.98	1	0.88
26	0.88	0.79	0.83	0.84	0.74	0.79	0.93	0.85	0.87
27	0.88	0.85	0.77	0.84	0.89	0.71	0.91	0.85	0.85
28	0.89	0.87	0.75	0.89	0.81	0.79	0.89	0.81	0.81
29	0.98	0.94	0.94	0.98	0.93	0.94	0.99	0.96	0.96
30	0.96	0.92	0.92	0.95	0.93	0.9	0.97	0.96	0.94
31	0.97	0.96	0.9	0.97	0.96	0.9	0.97	0.96	0.92
32	0.95	0.87	0.88	0.94	0.85	0.88	0.96	0.88	0.92
33	0.91	0.81	0.9	0.92	0.81	0.9	0.9	0.81	0.9
34	0.98	0.91	0.94	0.97	0.96	0.85	0.99	0.96	0.94
35	0.97	0.92	0.87	0.96	0.93	0.85	0.98	0.96	0.87
36	0.87	0.81	0.81	0.88	0.89	0.79	0.86	0.77	0.85
37	0.85	0.79	0.75	0.83	0.74	0.75	0.87	0.85	0.75
38	0.86	0.74	0.81	0.87	0.74	0.81	0.85	0.73	0.81
39	0.97	0.92	0.92	0.96	1	0.87	0.97	0.92	0.96
40	0.95	0.91	0.88	0.94	0.96	0.83	0.96	0.96	0.88
41	0.78	0.66	0.71	0.79	0.74	0.75	0.76	0.73	0.62
42	0.7	0.74	0.62	0.71	0.7	0.69	0.69	0.73	0.62
43	0.67	0.6	0.67	0.68	0.67	0.67	0.66	0.62	0.62
44	0.79	0.7	0.79	0.8	0.81	0.73	0.78	0.65	0.81
45	0.75	0.7	0.69	0.75	0.67	0.71	0.75	0.73	0.69
46	0.59	0.53	0.65	0.62	0.59	0.71	0.55	0.62	0.46
47	0.58	0.74	0.46	0.61	0.78	0.48	0.55	0.54	0.58
48	0.62	0.57	0.63	0.62	0.63	0.63	0.62	0.5	0.79
49	0.8	0.66	0.83	0.79	0.63	0.83	0.82	0.69	0.83
50	0.73	0.72	0.65	0.73	0.63	0.75	0.72	0.69	0.69
51	0.97	0.96	0.9	0.96	0.96	0.9	0.97	0.92	0.94
52	0.97	0.92	0.9	0.96	0.89	0.9	0.98	0.96	0.9
53	0.87	0.79	0.83	0.88	0.81	0.83	0.85	0.77	0.83

54	0.98	0.96	0.9	0.97	0.93	0.9	0.99	0.92	0.96
55	0.97	0.89	0.94	0.96	0.85	0.94	0.98	0.92	0.94
56	0.9	0.85	0.79	0.88	0.81	0.79	0.92	0.81	0.88
57	0.87	0.81	0.81	0.85	0.85	0.75	0.89	0.88	0.81
58	0.84	0.75	0.75	0.82	0.74	0.73	0.86	0.81	0.79
59	0.97	1	0.9	0.97	1	0.9	0.98	0.96	0.94
60	0.94	0.94	0.87	0.93	0.93	0.85	0.95	0.92	0.92
61	0.96	0.92	0.9	0.96	1	0.9	0.95	0.85	0.92
62	0.94	0.91	0.88	0.94	0.89	0.88	0.95	0.92	0.88
63	0.81	0.7	0.81	0.83	0.78	0.83	0.78	0.62	0.81
64	0.97	0.91	0.92	0.96	0.93	0.9	0.97	0.92	0.92
65	0.95	0.98	0.85	0.95	0.93	0.87	0.96	1	0.85
66	0.84	0.83	0.83	0.86	0.89	0.83	0.82	0.77	0.83
67	0.82	0.77	0.77	0.78	0.7	0.77	0.86	0.81	0.81
68	0.76	0.7	0.77	0.75	0.74	0.73	0.77	0.69	0.77
69	0.96	0.94	0.88	0.96	0.96	0.88	0.95	0.92	0.88
70	0.93	0.91	0.87	0.92	0.89	0.87	0.94	0.88	0.9
71	0.92	0.81	0.87	0.92	0.81	0.9	0.91	0.81	0.87
72	0.89	0.81	0.83	0.89	0.81	0.83	0.89	0.88	0.77
73	0.9	0.77	0.88	0.9	0.85	0.83	0.91	0.77	0.92
74	0.93	0.85	0.88	0.94	0.89	0.88	0.93	0.81	0.88
75	0.89	0.79	0.83	0.9	0.81	0.83	0.89	0.77	0.87
76	0.78	0.77	0.65	0.8	0.81	0.63	0.77	0.77	0.65
77	0.78	0.74	0.75	0.79	0.78	0.75	0.78	0.69	0.75
78	0.85	0.66	0.9	0.88	0.78	0.9	0.82	0.69	0.73
79	0.95	0.87	0.9	0.96	0.89	0.9	0.94	0.92	0.85
80	0.87	0.85	0.75	0.88	0.85	0.81	0.86	0.81	0.75
81	0.78	0.81	0.63	0.79	0.7	0.75	0.78	0.85	0.63
82	0.75	0.66	0.67	0.75	0.63	0.73	0.75	0.65	0.67
83	0.69	0.7	0.56	0.69	0.74	0.52	0.7	0.54	0.88
84	0.8	0.74	0.73	0.82	0.78	0.75	0.79	0.69	0.73
85	0.76	0.7	0.65	0.75	0.63	0.71	0.78	0.73	0.65
86	0.59	0.62	0.56	0.63	0.74	0.56	0.54	0.5	0.56
87	0.6	0.66	0.56	0.58	0.63	0.56	0.62	0.69	0.56
88	0.64	0.7	0.52	0.62	0.56	0.65	0.66	0.77	0.52
89	0.8	0.72	0.73	0.8	0.74	0.73	0.8	0.88	0.62
90	0.72	0.79	0.6	0.7	0.74	0.6	0.74	0.85	0.6
91	0.83	0.77	0.79	0.8	0.74	0.79	0.87	0.81	0.79
92	0.79	0.87	0.71	0.79	0.85	0.73	0.78	0.85	0.71
93	0.91	0.89	0.87	0.92	0.89	0.87	0.9	0.88	0.87
94	0.83	0.87	0.67	0.82	0.89	0.67	0.84	0.73	0.83
95	0.77	0.83	0.63	0.76	0.78	0.62	0.79	0.92	0.63
96	0.78	0.79	0.67	0.8	0.89	0.67	0.76	0.81	0.6
97	0.82	0.79	0.73	0.82	0.85	0.71	0.81	0.77	0.73
98	0.89	0.81	0.79	0.9	0.78	0.88	0.89	0.85	0.79
99	0.84	0.79	0.77	0.83	0.85	0.75	0.84	0.92	0.69
100	0.8	0.77	0.77	0.8	0.74	0.77	0.8	0.81	0.77

[a]No. referred to the number of the inter-replication experiment; there were 100 inter-replication combinations in total.

[b]CHD/MI/non-MI referred to the diagnostic performances in distinguishing CHD/MI/non-MI patients from HCs, as determined using the area under curve (AUC), sensitivity (Sen), and specificity (Spe).

Table S8. Diagnostic performance (no stabilization)for the discovery cohort with 100 inter-replication combinations.

No.[a]	CHD[b]			MI[b]			non-MI[b]		
	AUC	Sen	Spe	AUC	Sen	Spe	AUC	Sen	Spe
1	0.98	0.95	0.92	0.98	0.95	0.92	0.98	0.96	0.92
2	0.97	0.91	0.88	0.97	0.93	0.88	0.96	0.85	0.94
3	0.9	0.86	0.77	0.89	0.84	0.77	0.91	0.88	0.77
4	0.98	0.93	0.95	0.98	0.93	0.95	0.99	0.92	0.95
5	0.98	0.96	0.91	0.98	0.96	0.91	0.98	0.94	0.93
6	0.9	0.89	0.79	0.91	0.85	0.84	0.89	0.9	0.79
7	0.88	0.78	0.78	0.89	0.8	0.78	0.88	0.76	0.78
8	0.91	0.84	0.84	0.91	0.85	0.84	0.9	0.86	0.83
9	0.98	0.94	0.96	0.98	0.94	0.96	0.98	0.94	0.97
10	0.95	0.91	0.88	0.96	0.93	0.88	0.95	0.9	0.88
11	0.95	0.88	0.9	0.94	0.85	0.91	0.97	0.91	0.9
12	0.94	0.87	0.87	0.93	0.84	0.87	0.95	0.9	0.87
13	0.89	0.78	0.86	0.88	0.75	0.88	0.89	0.82	0.84
14	0.96	0.88	0.91	0.95	0.86	0.89	0.98	0.91	0.91
15	0.96	0.89	0.89	0.94	0.86	0.88	0.97	0.92	0.91
16	0.85	0.75	0.8	0.85	0.75	0.85	0.85	0.74	0.8
17	0.85	0.76	0.77	0.84	0.78	0.77	0.86	0.72	0.86
18	0.88	0.79	0.81	0.87	0.75	0.81	0.89	0.84	0.81
19	0.96	0.88	0.91	0.95	0.85	0.91	0.97	0.9	0.93
20	0.92	0.84	0.86	0.91	0.85	0.84	0.93	0.85	0.89
21	0.98	0.95	0.96	0.98	0.96	0.96	0.98	0.96	0.94
22	0.98	0.92	0.93	0.98	0.95	0.93	0.98	0.9	0.92
23	0.95	0.88	0.88	0.95	0.87	0.88	0.96	0.88	0.88
24	0.99	0.96	0.94	0.99	0.95	0.94	0.99	0.96	0.96
25	0.98	0.95	0.91	0.98	0.97	0.9	0.99	0.96	0.93
26	0.92	0.86	0.8	0.93	0.83	0.85	0.91	0.88	0.78
27	0.91	0.83	0.83	0.92	0.85	0.83	0.91	0.81	0.83
28	0.95	0.89	0.89	0.94	0.89	0.89	0.95	0.94	0.87
29	0.99	0.97	0.93	0.99	0.98	0.93	0.99	0.92	0.96
30	0.96	0.94	0.89	0.97	0.95	0.9	0.95	0.92	0.88
31	0.99	0.95	0.94	0.99	0.95	0.95	0.99	0.95	0.94
32	0.98	0.93	0.92	0.99	0.96	0.92	0.97	0.94	0.9
33	0.94	0.84	0.88	0.94	0.87	0.88	0.93	0.89	0.81
34	0.99	0.97	0.96	0.99	0.98	0.96	0.99	0.98	0.95
35	0.99	0.96	0.93	0.99	0.95	0.94	0.99	0.96	0.93
36	0.92	0.8	0.86	0.93	0.81	0.9	0.91	0.78	0.85
37	0.92	0.8	0.84	0.93	0.84	0.84	0.9	0.81	0.8
38	0.94	0.86	0.85	0.94	0.85	0.87	0.94	0.87	0.85
39	0.99	0.97	0.93	0.99	0.99	0.93	0.99	0.94	0.96
40	0.97	0.92	0.89	0.97	0.92	0.91	0.96	0.92	0.87
41	0.87	0.79	0.78	0.85	0.74	0.81	0.89	0.85	0.78
42	0.85	0.8	0.75	0.84	0.75	0.77	0.86	0.84	0.75
43	0.8	0.68	0.77	0.78	0.68	0.73	0.83	0.71	0.82
44	0.88	0.79	0.79	0.87	0.85	0.72	0.9	0.83	0.79
45	0.87	0.82	0.78	0.85	0.81	0.78	0.89	0.84	0.78
46	0.75	0.8	0.63	0.75	0.81	0.63	0.74	0.78	0.63
47	0.72	0.76	0.59	0.7	0.75	0.59	0.73	0.73	0.62
48	0.79	0.73	0.71	0.78	0.73	0.71	0.8	0.72	0.72
49	0.88	0.78	0.81	0.87	0.75	0.79	0.89	0.85	0.81
50	0.82	0.79	0.7	0.82	0.77	0.7	0.83	0.83	0.7
51	0.98	0.96	0.91	0.98	0.95	0.92	0.98	0.96	0.91
52	0.98	0.94	0.89	0.98	0.95	0.9	0.97	0.91	0.89
53	0.93	0.83	0.88	0.94	0.86	0.86	0.93	0.82	0.88

54	0.99	0.96	0.97	0.99	0.95	0.97	0.99	0.96	0.97
55	0.99	0.94	0.94	0.99	0.95	0.94	0.99	0.96	0.93
56	0.92	0.84	0.82	0.93	0.84	0.83	0.91	0.85	0.82
57	0.91	0.82	0.8	0.91	0.84	0.8	0.9	0.78	0.84
58	0.93	0.86	0.84	0.94	0.85	0.87	0.92	0.87	0.82
59	0.99	0.94	0.96	0.99	0.94	0.97	0.99	0.94	0.96
60	0.96	0.87	0.91	0.97	0.89	0.91	0.96	0.87	0.89
61	0.97	0.95	0.9	0.97	0.95	0.91	0.97	0.96	0.9
62	0.97	0.92	0.88	0.98	0.9	0.92	0.96	0.9	0.88
63	0.92	0.84	0.85	0.92	0.83	0.85	0.93	0.86	0.85
64	0.99	0.94	0.95	0.99	0.94	0.96	0.99	0.95	0.95
65	0.98	0.93	0.94	0.98	0.94	0.95	0.98	0.96	0.92
66	0.9	0.82	0.78	0.91	0.77	0.89	0.89	0.81	0.78
67	0.9	0.86	0.76	0.92	0.84	0.8	0.88	0.85	0.75
68	0.91	0.84	0.82	0.92	0.82	0.85	0.9	0.83	0.82
69	0.98	0.93	0.93	0.98	0.92	0.94	0.98	0.94	0.93
70	0.96	0.87	0.9	0.96	0.88	0.9	0.95	0.86	0.91
71	0.95	0.9	0.85	0.95	0.88	0.88	0.94	0.91	0.84
72	0.93	0.86	0.87	0.94	0.9	0.85	0.93	0.84	0.87
73	0.94	0.85	0.87	0.95	0.88	0.89	0.92	0.86	0.8
74	0.96	0.9	0.87	0.96	0.89	0.89	0.95	0.89	0.87
75	0.96	0.9	0.91	0.96	0.9	0.9	0.96	0.9	0.91
76	0.86	0.76	0.79	0.88	0.79	0.79	0.85	0.87	0.7
77	0.86	0.77	0.81	0.88	0.81	0.81	0.83	0.76	0.76
78	0.93	0.83	0.85	0.93	0.85	0.85	0.92	0.84	0.82
79	0.96	0.9	0.91	0.96	0.91	0.91	0.95	0.89	0.91
80	0.93	0.87	0.84	0.93	0.88	0.84	0.91	0.85	0.84
81	0.89	0.83	0.82	0.87	0.79	0.82	0.92	0.86	0.86
82	0.85	0.77	0.77	0.84	0.76	0.77	0.87	0.8	0.77
83	0.85	0.74	0.78	0.84	0.81	0.72	0.85	0.73	0.84
84	0.88	0.79	0.83	0.84	0.75	0.78	0.93	0.88	0.83
85	0.89	0.76	0.85	0.86	0.72	0.85	0.92	0.81	0.86
86	0.74	0.67	0.71	0.74	0.69	0.69	0.75	0.67	0.72
87	0.74	0.7	0.68	0.73	0.7	0.68	0.76	0.71	0.69
88	0.81	0.73	0.76	0.8	0.73	0.73	0.84	0.75	0.77
89	0.89	0.87	0.78	0.88	0.85	0.78	0.9	0.83	0.84
90	0.85	0.81	0.77	0.83	0.77	0.76	0.89	0.87	0.78
91	0.89	0.76	0.86	0.9	0.78	0.86	0.89	0.75	0.85
92	0.88	0.82	0.75	0.88	0.83	0.75	0.88	0.82	0.77
93	0.95	0.91	0.88	0.96	0.94	0.89	0.94	0.91	0.86
94	0.9	0.87	0.81	0.91	0.87	0.81	0.89	0.86	0.81
95	0.87	0.81	0.77	0.86	0.82	0.77	0.87	0.81	0.77
96	0.87	0.78	0.78	0.9	0.84	0.79	0.84	0.82	0.72
97	0.84	0.75	0.75	0.87	0.79	0.8	0.81	0.74	0.69
98	0.94	0.9	0.8	0.94	0.89	0.81	0.93	0.85	0.85
99	0.92	0.85	0.84	0.91	0.89	0.8	0.92	0.87	0.84
100	0.85	0.84	0.73	0.85	0.87	0.73	0.84	0.81	0.73

[a]No. referred to the number of the inter-replication experiment; there were 100 inter-replication combinations in total.

[b]CHD/MI/non-MI referred to the diagnostic performance in distinguishing CHD/MI/non-MI patients from HCs, as determined using the area under curve (AUC), sensitivity (Sen), and specificity (Spe).

Table S9. Quantitative results using the deep stabilizer under different configurations.

With attention[a]	n_f [b]	ℓ [c]	$Blocks$ [d]	Params[e]	PSNR[f] (dB)	Diagnostic performance[g]
No	32	3	4	200k	25.30	0.94 ± 0.03
Yes	32	3	4	200k	25.89	0.95 ± 0.03
Yes	32	4	4	292k	25.48	0.94± 0.04
Yes	32	3	5	708k	24.94	0.95± 0.03
Yes	48	3	4	448k	25.70	0.95 ± 0.03

[a]With attention indicated whether spectra were stabilized with an attention mechanism.

[b]n_f referred to the filter numbers in convolution operations.

[c]ℓ referred to the depth of dense blocks.

[d]$Blocks$ referred to the number of dense blocks.

[e]Params referred to the number of parameters in the generator.

[f]PSNR referred to the peak signal-to-noise ratio for the stabilized spectra and reference spectra.

[g]Diagnostic performance (deep-stabilized) referred to the AUC for the diagnosis of CHD.

Table S10. Numbers of peaks selected by the attention mechanism andS/N.

Sample No.[a]	Replicate No.[b]	Attention[c]	S/N[d]	Intersection[e]
1-261	1	741	92	84
	2	576	127	115
	3	597	111	99
	4	349	75	70
	5	755	133	121
	6	786	112	101
	7	436	87	81
	8	690	122	111
	9	388	80	71
	10	622	121	107
262-517	1	687	142	131
	2	386	98	89
	3	454	93	85
	4	698	125	114
	5	703	136	122
	6	476	122	109
	7	558	155	141
	8	367	121	111
	9	497	105	94
	10	576	104	93

[a]Sample No. referred to the sample numbers in this study.

[b]Replicate No. referred to the number of the technical replicate. Ten independent technical replicates were conducted to obtain MS spectra with 10,000 laser shots.

[c]Attention referred to the number of peaks selected by the attention mechanism based on a threshold of 0.5.

[d]S/N referred to the number of peaks selected according to signal/noise ratio (S/N) based on a threshold of 3.

[e]Intersection referred to the number of peaks in the intersection of peaks selected by the attention mechanism and S/N.

Table S11. Diagnostic performance (deep-stabilized) for the validation cohort with 100 inter-replication combinations.

No.[a]	CHD[b]			MI[b]			non-MI[b]		
	AUC	Sen	Spe	AUC	Sen	Spe	AUC	Sen	Spe
1	0.94	0.94	0.87	0.93	0.96	0.85	0.94	1.00	0.87
2	0.98	0.94	0.96	0.97	0.89	0.96	0.98	1.00	0.96
3	0.98	0.89	0.98	0.98	0.89	0.98	0.98	0.88	0.98
4	0.96	0.98	0.88	0.96	1.00	0.88	0.95	0.96	0.88
5	0.98	0.94	0.90	0.98	0.96	0.90	0.98	1.00	0.88
6	0.92	0.85	0.87	0.90	0.93	0.77	0.95	0.96	0.87
7	0.90	0.89	0.85	0.92	0.93	0.85	0.89	0.85	0.85
8	0.95	0.87	0.85	0.96	0.89	0.92	0.93	0.88	0.79
9	0.96	0.94	0.88	0.96	0.96	0.88	0.96	0.92	0.90
10	0.94	0.89	0.88	0.92	0.89	0.85	0.96	1.00	0.88
11	0.94	0.92	0.85	0.93	0.96	0.83	0.94	0.96	0.85
12	0.99	0.94	0.96	0.99	0.93	0.96	0.99	0.96	0.96
13	0.96	0.91	0.92	0.95	0.89	0.92	0.97	0.92	0.92
14	0.97	0.92	0.90	0.97	0.93	0.90	0.98	0.96	0.88
15	0.98	0.92	0.94	0.98	0.93	0.94	0.98	0.92	0.96
16	0.90	0.92	0.81	0.87	0.85	0.81	0.94	0.96	0.87
17	0.91	0.92	0.79	0.88	0.85	0.79	0.94	0.92	0.83
18	0.95	0.91	0.85	0.97	0.89	1.00	0.93	0.85	0.85
19	0.98	0.96	0.92	0.98	0.96	0.90	0.98	0.96	0.94
20	0.95	0.87	0.87	0.95	0.93	0.87	0.94	0.96	0.81
21	0.97	0.98	0.88	0.97	0.96	0.90	0.97	1.00	0.88
22	1.00	0.98	0.98	1.00	0.96	1.00	1.00	1.00	0.98
23	0.99	0.92	0.98	0.99	0.93	0.98	0.99	0.92	0.98
24	0.99	1.00	0.92	0.99	1.00	0.92	0.99	1.00	0.92
25	0.99	0.96	0.96	0.99	0.96	0.96	0.99	0.96	0.98
26	0.95	0.87	0.87	0.94	0.85	0.87	0.96	0.88	0.96
27	0.93	0.91	0.87	0.93	0.93	0.87	0.93	0.88	0.87
28	0.94	0.89	0.85	0.95	0.96	0.85	0.93	0.88	0.81
29	0.99	0.98	0.92	0.99	1.00	0.92	0.98	0.96	0.92
30	0.97	1.00	0.90	0.97	1.00	0.90	0.97	1.00	0.90
31	0.93	0.91	0.85	0.92	0.93	0.83	0.94	0.92	0.85
32	0.98	0.92	0.92	0.98	0.96	0.88	0.99	0.96	0.92
33	0.98	0.91	0.94	0.98	0.93	0.94	0.98	0.92	0.90
34	0.96	0.96	0.85	0.95	0.93	0.85	0.97	0.92	0.88
35	0.98	0.91	0.92	0.97	0.93	0.87	0.99	0.96	0.94
36	0.92	0.89	0.81	0.90	0.89	0.79	0.94	0.96	0.81
37	0.91	0.91	0.81	0.90	0.93	0.81	0.92	0.85	0.87
38	0.93	0.85	0.87	0.94	0.89	0.87	0.91	0.81	0.87
39	0.96	1.00	0.85	0.96	0.93	0.88	0.96	1.00	0.85
40	0.94	0.87	0.87	0.92	0.89	0.83	0.96	0.88	0.90
41	0.92	0.94	0.83	0.93	0.96	0.83	0.91	0.92	0.83
42	0.97	0.92	0.90	0.95	0.89	0.88	0.98	1.00	0.94
43	0.94	0.85	0.92	0.90	0.74	0.94	0.98	0.96	0.92
44	0.95	0.92	0.90	0.95	0.93	0.88	0.95	0.96	0.90
45	0.97	0.92	0.88	0.96	0.96	0.85	0.99	0.92	0.96
46	0.90	0.91	0.79	0.87	0.89	0.79	0.93	0.92	0.83
47	0.87	0.85	0.75	0.85	0.89	0.73	0.89	0.85	0.83
48	0.87	0.87	0.75	0.87	0.78	0.87	0.87	0.85	0.79
49	0.96	0.96	0.90	0.95	0.93	0.90	0.97	0.96	0.92
50	0.94	0.85	0.92	0.92	0.93	0.81	0.95	0.92	0.92
51	0.96	1.00	0.90	0.97	1.00	0.90	0.96	1.00	0.90
52	1.00	1.00	0.98	1.00	1.00	0.98	0.99	1.00	0.98
53	1.00	0.96	0.96	1.00	0.96	1.00	1.00	0.96	0.96

54	0.98	0.98	0.92	0.98	1.00	0.92	0.98	0.96	0.92
55	0.99	1.00	0.90	0.98	1.00	0.90	0.99	0.92	0.94
56	0.96	0.96	0.87	0.95	0.93	0.87	0.98	0.96	0.92
57	0.94	0.87	0.90	0.93	0.85	0.90	0.95	0.88	0.90
58	0.96	0.92	0.87	0.97	0.96	0.92	0.94	0.88	0.87
59	0.96	0.94	0.92	0.98	1.00	0.92	0.94	0.96	0.90
60	0.96	0.96	0.90	0.96	0.96	0.90	0.96	0.96	0.90
61	0.97	0.91	0.90	0.97	1.00	0.87	0.97	0.92	0.90
62	0.99	0.98	0.96	1.00	1.00	0.96	0.99	0.96	0.96
63	0.98	0.94	0.92	0.98	0.96	0.92	0.98	0.92	0.96
64	0.98	0.96	0.90	0.97	0.96	0.90	0.98	0.96	0.90
65	0.98	0.96	0.92	0.98	0.96	0.92	0.99	0.96	0.98
66	0.94	0.92	0.83	0.92	0.93	0.79	0.96	0.88	0.90
67	0.92	0.87	0.81	0.92	0.89	0.81	0.92	0.81	0.92
68	0.91	0.83	0.85	0.92	0.89	0.83	0.90	0.88	0.79
69	0.97	0.91	0.96	0.98	0.96	0.96	0.96	0.92	0.88
70	0.96	0.92	0.90	0.97	0.93	0.90	0.96	0.92	0.90
71	0.92	0.87	0.87	0.91	0.85	0.81	0.94	0.96	0.87
72	0.98	0.96	0.92	0.98	0.93	0.94	0.99	1.00	0.92
73	0.98	0.91	0.94	0.97	0.93	0.88	0.98	0.96	0.94
74	0.96	0.94	0.88	0.95	0.93	0.88	0.96	0.96	0.88
75	0.96	0.91	0.92	0.95	0.89	0.92	0.98	0.92	0.92
76	0.88	0.85	0.79	0.88	0.89	0.79	0.88	0.92	0.75
77	0.90	0.92	0.81	0.89	0.93	0.81	0.91	0.92	0.83
78	0.93	0.87	0.90	0.95	0.96	0.90	0.91	0.88	0.83
79	0.96	0.91	0.90	0.96	0.93	0.88	0.96	0.92	0.90
80	0.93	0.87	0.87	0.92	0.85	0.88	0.94	0.92	0.85
81	0.92	0.87	0.88	0.90	0.81	0.88	0.93	0.92	0.88
82	0.98	0.92	0.94	0.98	0.93	0.94	0.98	0.92	0.96
83	0.99	0.96	0.96	0.98	0.93	0.96	1.00	1.00	0.96
84	0.92	0.91	0.81	0.91	0.89	0.81	0.93	0.92	0.83
85	0.94	0.91	0.87	0.92	0.93	0.83	0.96	0.96	0.87
86	0.88	0.87	0.81	0.87	0.89	0.81	0.90	0.96	0.77
87	0.88	0.85	0.88	0.89	0.85	0.88	0.86	0.85	0.90
88	0.92	0.83	0.90	0.94	0.93	0.90	0.90	0.88	0.77
89	0.94	0.89	0.83	0.94	1.00	0.77	0.94	0.96	0.83
90	0.91	0.87	0.87	0.91	0.89	0.87	0.91	0.85	0.87
91	0.95	0.92	0.87	0.95	0.93	0.87	0.94	0.92	0.88
92	0.95	0.91	0.92	0.98	0.96	0.92	0.93	0.85	0.92
93	0.97	0.91	0.96	0.97	0.93	0.98	0.97	0.96	0.88
94	0.95	0.92	0.92	0.96	0.96	0.92	0.94	0.88	0.92
95	0.94	0.91	0.83	0.95	0.85	0.88	0.94	0.92	0.81
96	0.93	0.85	0.87	0.94	0.93	0.87	0.91	0.92	0.77
97	0.90	0.77	0.85	0.89	0.89	0.73	0.91	0.81	0.90
98	0.94	0.81	0.92	0.96	0.89	0.92	0.91	0.81	0.83
99	0.95	0.94	0.87	0.96	0.93	0.90	0.94	0.92	0.87
100	0.93	0.91	0.83	0.93	0.93	0.83	0.92	0.88	0.83

[a]No. referred to the number of the inter-replication experiment; there were 100 inter-replication combinations in total.

[b]CHD/MI/non-MI referred to the diagnostic performance in distinguishing CHD/MI/non-MI patients from HCs, as determined by the area under curve (AUC), sensitivity (Sen), and specificity (Spe).

Table S12. Diagnostic performance (deep-stabilized) for the discovery cohort with 100 inter-replication combinations.

No.[a]	CHD[b]			MI[b]			non-MI[b]		
	AUC	Sen	Spe	AUC	Sen	Spe	AUC	Sen	Spe
1	0.96	0.95	0.87	0.96	0.97	0.87	0.95	0.95	0.87
2	0.98	0.94	0.92	0.98	0.95	0.92	0.98	0.92	0.92
3	0.99	0.95	0.95	0.99	0.96	0.95	0.99	0.95	0.94
4	0.97	0.96	0.89	0.97	0.94	0.90	0.97	0.98	0.89
5	0.98	0.96	0.91	0.99	0.95	0.92	0.98	0.97	0.90
6	0.93	0.86	0.85	0.94	0.88	0.85	0.92	0.91	0.80
7	0.93	0.88	0.82	0.93	0.90	0.82	0.92	0.90	0.79
8	0.95	0.88	0.88	0.96	0.89	0.92	0.93	0.84	0.88
9	0.98	0.94	0.92	0.98	0.96	0.90	0.98	0.95	0.93
10	0.96	0.94	0.88	0.97	0.90	0.92	0.95	0.94	0.88
11	0.96	0.93	0.89	0.96	0.94	0.89	0.95	0.92	0.89
12	0.97	0.92	0.90	0.97	0.92	0.90	0.97	0.96	0.88
13	0.99	0.93	0.96	0.99	0.94	0.97	0.99	0.96	0.92
14	0.97	0.92	0.90	0.97	0.90	0.90	0.98	0.91	0.93
15	0.98	0.92	0.91	0.97	0.91	0.91	0.98	0.92	0.93
16	0.94	0.89	0.83	0.94	0.94	0.83	0.93	0.91	0.79
17	0.93	0.88	0.82	0.93	0.91	0.82	0.92	0.85	0.82
18	0.94	0.86	0.88	0.95	0.90	0.92	0.92	0.90	0.80
19	0.98	0.93	0.92	0.98	0.93	0.92	0.98	0.96	0.91
20	0.95	0.90	0.89	0.95	0.95	0.86	0.95	0.91	0.89
21	0.97	0.94	0.89	0.97	0.95	0.89	0.97	0.92	0.89
22	0.98	0.96	0.91	0.98	0.95	0.91	0.98	0.97	0.91
23	0.98	0.94	0.94	0.99	0.97	0.94	0.98	0.96	0.89
24	0.98	0.92	0.94	0.98	0.96	0.90	0.99	0.96	0.94
25	0.99	0.97	0.94	0.99	0.96	0.93	0.99	0.98	0.94
26	0.94	0.95	0.82	0.95	0.92	0.83	0.94	0.94	0.83
27	0.93	0.93	0.81	0.94	0.95	0.81	0.92	0.91	0.81
28	0.95	0.87	0.89	0.96	0.88	0.93	0.93	0.84	0.89
29	0.99	0.96	0.92	0.99	0.94	0.95	0.98	0.95	0.92
30	0.96	0.90	0.89	0.96	0.91	0.89	0.95	0.89	0.89
31	0.96	0.96	0.88	0.97	0.97	0.89	0.96	0.94	0.88
32	0.98	0.91	0.93	0.98	0.93	0.91	0.97	0.91	0.93
33	0.99	0.96	0.95	0.99	0.95	0.96	0.99	0.96	0.95
34	0.98	0.97	0.92	0.98	0.95	0.93	0.98	0.98	0.92
35	0.98	0.96	0.91	0.98	0.93	0.94	0.98	0.96	0.91
36	0.94	0.91	0.82	0.95	0.89	0.86	0.93	0.89	0.82
37	0.93	0.93	0.79	0.94	0.95	0.79	0.92	0.95	0.78
38	0.94	0.82	0.95	0.96	0.87	0.95	0.93	0.83	0.84
39	0.98	0.96	0.92	0.99	0.94	0.94	0.98	0.96	0.92
40	0.96	0.94	0.87	0.96	0.95	0.87	0.95	0.91	0.88
41	0.94	0.89	0.88	0.94	0.92	0.85	0.95	0.92	0.88
42	0.96	0.90	0.89	0.95	0.91	0.89	0.96	0.91	0.89
43	0.97	0.89	0.93	0.97	0.88	0.93	0.97	0.91	0.92
44	0.96	0.92	0.90	0.95	0.92	0.89	0.96	0.95	0.90
45	0.97	0.92	0.92	0.96	0.90	0.92	0.97	0.94	0.92
46	0.90	0.86	0.77	0.90	0.92	0.74	0.89	0.84	0.80
47	0.92	0.86	0.80	0.92	0.90	0.80	0.91	0.92	0.76
48	0.90	0.78	0.89	0.90	0.79	0.89	0.90	0.76	0.89
49	0.97	0.92	0.90	0.97	0.91	0.89	0.97	0.92	0.93
50	0.94	0.90	0.83	0.95	0.86	0.87	0.93	0.89	0.83
51	0.96	0.95	0.88	0.97	0.95	0.88	0.95	0.94	0.88
52	0.98	0.95	0.90	0.98	0.95	0.92	0.97	0.94	0.90
53	0.99	0.94	0.93	0.99	0.95	0.97	0.98	0.92	0.91

54	0.98	0.97	0.93	0.98	0.96	0.93	0.98	0.97	0.93
55	0.99	0.95	0.94	0.99	0.94	0.95	0.99	0.96	0.94
56	0.94	0.91	0.81	0.95	0.91	0.83	0.92	0.90	0.81
57	0.93	0.92	0.80	0.94	0.90	0.83	0.91	0.94	0.79
58	0.94	0.85	0.86	0.95	0.86	0.92	0.92	0.85	0.81
59	0.98	0.97	0.90	0.99	0.94	0.95	0.97	0.95	0.90
60	0.96	0.93	0.87	0.96	0.94	0.87	0.95	0.87	0.91
61	0.97	0.90	0.91	0.97	0.95	0.91	0.96	0.96	0.86
62	0.98	0.93	0.92	0.98	0.95	0.92	0.97	0.94	0.90
63	0.99	0.96	0.94	0.99	0.95	0.97	0.99	0.98	0.93
64	0.98	0.94	0.94	0.99	0.95	0.93	0.98	0.95	0.94
65	0.98	0.96	0.92	0.99	0.95	0.94	0.98	0.92	0.92
66	0.94	0.91	0.83	0.95	0.91	0.85	0.93	0.92	0.81
67	0.93	0.90	0.81	0.93	0.93	0.81	0.92	0.94	0.78
68	0.93	0.85	0.87	0.95	0.88	0.92	0.92	0.81	0.85
69	0.98	0.94	0.94	0.99	0.95	0.96	0.98	0.92	0.93
70	0.96	0.92	0.87	0.97	0.90	0.90	0.96	0.94	0.86
71	0.96	0.94	0.88	0.97	0.91	0.91	0.96	0.92	0.88
72	0.97	0.96	0.90	0.98	0.97	0.90	0.97	0.97	0.89
73	0.99	0.96	0.96	0.99	0.96	0.97	0.99	0.95	0.96
74	0.98	0.96	0.89	0.97	0.97	0.89	0.98	0.96	0.90
75	0.99	0.94	0.92	0.99	0.95	0.92	0.99	0.92	0.95
76	0.92	0.83	0.82	0.94	0.87	0.82	0.90	0.85	0.77
77	0.93	0.90	0.80	0.94	0.89	0.84	0.92	0.91	0.79
78	0.95	0.86	0.89	0.96	0.87	0.96	0.93	0.81	0.89
79	0.98	0.94	0.92	0.98	0.96	0.91	0.98	0.94	0.92
80	0.96	0.89	0.88	0.96	0.90	0.89	0.95	0.87	0.88
81	0.95	0.96	0.84	0.96	0.93	0.86	0.95	0.94	0.83
82	0.97	0.94	0.87	0.97	0.89	0.93	0.96	0.88	0.90
83	0.99	0.93	0.97	0.99	0.96	0.98	0.99	0.96	0.90
84	0.97	0.92	0.91	0.97	0.95	0.88	0.97	0.94	0.91
85	0.97	0.94	0.92	0.98	0.95	0.92	0.97	0.92	0.92
86	0.91	0.83	0.82	0.93	0.85	0.83	0.89	0.78	0.82
87	0.91	0.88	0.83	0.92	0.90	0.83	0.90	0.87	0.82
88	0.95	0.87	0.89	0.96	0.90	0.89	0.93	0.90	0.83
89	0.98	0.93	0.92	0.98	0.95	0.92	0.97	0.90	0.91
90	0.95	0.89	0.88	0.96	0.91	0.89	0.93	0.90	0.83
91	0.96	0.90	0.90	0.96	0.92	0.90	0.96	0.97	0.86
92	0.96	0.92	0.89	0.96	0.94	0.88	0.96	0.90	0.89
93	0.99	0.94	0.96	0.99	0.96	0.96	0.98	0.94	0.94
94	0.96	0.95	0.88	0.96	0.94	0.89	0.96	0.95	0.88
95	0.97	0.93	0.90	0.97	0.92	0.90	0.98	0.95	0.90
96	0.94	0.92	0.85	0.95	0.90	0.87	0.93	0.92	0.85
97	0.93	0.88	0.86	0.93	0.88	0.85	0.93	0.88	0.86
98	0.95	0.88	0.89	0.96	0.91	0.89	0.93	0.86	0.87
99	0.97	0.94	0.91	0.98	0.96	0.91	0.97	0.95	0.89
100	0.95	0.92	0.88	0.95	0.94	0.88	0.94	0.90	0.88

[a]No. referred to the number of the inter-replication experiment; there were 100 inter-replication combinations in total.

[b]CHD/MI/non-MI referred to the diagnostic performance in distinguishing CHD/MI/non-MI patients from HCs, as determined by the area under curve (AUC), sensitivity (Sen), and specificity (Spe).

Table S13. Newly screened m/z features with the deep stabilizer.

Metabolite	HMDB ID	p value[a]	p value[b]	Fre[c]	Int[d]	CHD diff [e]
Lactic acid (LA)	HMDB0000190	5.23E-01	2.81E-14	100	3253	↑
Methylimidazoleacetic acid (MIAA)	HMDB0002820	3.93E-01	7.62E-06	100	89	↑
cis-Aconitic acid (cis-AC)	HMDB0000072	8.30E-01	1.55E-06	83	57	↑
Diacylglycerol (14:1/24:1) (DAG-1)	HMDB0007065	6.45E-02	3.71E-04	99	36	↓
Diacylglycerol (24:1/20:4) (DAG-2)	HMDB0007838	4.70E-02	5.59E-10	100	30	↓

[a]p value referred to the p value obtained from an independent-samples t-test of a single m/z feature of no stabilization MS data between CHD patients and controls.

[b]p value referred to the p value obtained from an independent-samples t-test of a single m/z feature of deep-stabilized MS data between CHD patients and controls.

[c]Fre referred to the hit frequency of a single m/z feature of deep-stabilized MS data, which was selected from 100 sparse learning models as a screened biomarker.

[d]Int referred to the mean intensity at a single m/z feature of deep-stabilized MS data.

[e]CHD diffreferredto whether the single m/z feature (deep-stabilized) was increased or decreased in intensity, in CHD patients compared to controls.
